# Systematic Phenotyping and Molecular Analysis of the Woozy Mouse: A Preclinical Model of Cerebellar Ataxia

**DOI:** 10.1007/s12035-025-05577-y

**Published:** 2025-12-06

**Authors:** Fabio Bellia, Laura Amodei, Anna Giulia Ruggieri, Francesca Potenza, Marianna Viele, Manuela Bomba, Francesco Del Pizzo, Manuela Iezzi, Alberto Granzotto, Luca Federici, Michele Sallese

**Affiliations:** 1 Center for Advanced Studies and Technology (CAST), “G. d’Annunzio” University of Chieti-Pescara, 66100 Chieti, Italy; 2 Department of Innovative Technologies in Medicine and Dentistry, “G. d’Annunzio” University of Chieti-Pescara, 66100 Chieti, Italy; 3 Department of Neuroscience, Imaging, and Clinical Sciences, “G. d’Annunzio” University of Chieti-Pescara, 66100 Chieti, Italy

**Keywords:** Coordination impairments, Sex-related differences, Brain functional disturbances, Rare genetic disorders

## Abstract

Over the years, a diverse range of animal models has been established and employed in both basic and translational research, elucidating the intricate molecular mechanisms that drive cerebellar ataxias. However, many preclinical models remain insufficiently characterised, hindering our understanding of the diseases and the development of effective therapeutic approaches. The present study provides a comprehensive phenotypic and molecular characterisation of the woozy (*Sil1*^*wz*^) mouse model of Marinesco-Sjögren Syndrome (MSS), a rare autosomal recessive cerebellar ataxia characterised by cerebellar dysfunction, congenital cataracts, and progressive myopathy. Disease progression was monitored from 5 to 26 weeks using a series of motor assessments, including the accelerating rotarod, beam walking, pole test, and inverted grid test. These evaluations revealed progressive cerebellar ataxia in *Sil1*^*wz*^ mice, with symptoms emerging around the ninth week of age. A clear sex-related difference was observed in the motor tests, with female *Sil1*^*wz*^ mice outperforming their male counterparts across all assessments. Histological analysis revealed significant muscular atrophy in glycolytic muscles (gastrocnemius and quadriceps) but not in oxidative muscles (soleus) of 26-week-old *Sil1*^*wz*^ mice. Molecular analyses confirmed the upregulation of unfolded protein response markers (BiP and pEIF2α) and proteolysis-associated proteins (Rab11 and LC3-II) in glycolytic but not oxidative muscles. Cognitive assessment using nesting behaviour showed deficits in *Sil1*^*wz*^ mice at 14 weeks, assuming similarities with mental retardation shown by MSS patients. Our findings established a comprehensive timeline of disease progression in this MSS model and highlighted the differential vulnerability of glycolytic versus oxidative muscles and cerebellar regions. These insights pave the way for the development of therapeutic strategies for MSS and related cerebellar ataxias.

**Supplementary Information:**

The online version contains supplementary material available at 10.1007/s12035-025-05577-y.

## Introduction

Autosomal recessive cerebellar ataxias (ARCA) represent a heterogeneous group of neurodegenerative disorders characterised by progressive cerebellar dysfunction, leading to impaired motor coordination, balance, and speech [1]. These conditions typically manifest in early childhood or adolescence, significantly impacting patients' quality of life and life expectancy [2]. Among the diverse spectrum of ARCAs, Marinesco-Sjögren Syndrome (MSS) stands out as a distinct clinical entity with unique pathophysiological features [3]. MSS, first described by Gheorghe Marinescu in 1931 [4] and later elaborated upon by Torsten Sjögren in 1950 [5], is characterised by a triad of clinical manifestations: cerebellar ataxia, congenital cataracts, and progressive myopathy [6–8]. This rare genetic disorder is caused by mutations in the *SIL1* gene in about 60% of cases [9], which encodes the nucleotide exchange factor SIL1, an essential component of the endoplasmic reticulum protein quality control machinery [10]. The loss of functional SIL1 leads to the accumulation of misfolded proteins, cellular stress, and, ultimately, neurodegeneration [11].

Due to the complexity of ARCA diseases, particularly MSS, designing and developing appropriate preclinical models for understanding disease processes and exploring well-defined therapeutic strategies is crucial. Such examples are the rodent models which reproduce the clinical and biochemical features of human subjects affected by Friedreich ataxia (FRDA). The FXN-MCK mouse is a well-characterised tissue-specific (Frataxin) *FXN*-knockout in cardiac and skeletal muscle [12], which gives the possibility to explore protein reintroduction in several ways [13–15].

Two available MSS preclinical models are the woozy (*Sil1*^*wz*^) and *Sil1*^*Gt*^ mouse. These two models were genetically selected for their potential to mimic a patient’s phenotype. The woozy mouse, which has a spontaneous *Sil1* gene mutation involving the insertion of an ETn retrotransposon between exons 7 and 8, has provided invaluable insights into how protein quality control functions in the cerebellum and neurodegeneration. The *Sil1*^*Gt*^ mouse is a genetically engineered mouse, developed using a β-geo gene-trap vector between exon 7 and 8 of the *Sil1* gene, resulting in truncation of the *Sil1* transcript after exon 7 [16]. Previous studies already highlighted the key features of the woozy and *Sil1*^*Gt*^ mouse models [16–20]. Clear symptoms of cerebellar ataxia were observed in these preclinical models, with mice showing Purkinje cell (PC) degeneration already during adolescence, around their 5/6^th^ week, ultimately losing these cells before the 3^rd^ month of life [16, 17, 19, 20]. General manifestations of muscle atrophy, with variation in fibre size, internalised nuclei, autophagic structures, and membrane-bound structures surrounding muscular cells nuclei similar to what was observed in MSS patients, were reported in these mice [18–24].

Despite the studies mentioned earlier, several characteristics of the woozy mouse remain unexplored, and it is still unclear how much they reflect all the clinical characteristics of MSS. However, these preclinical models currently face significant shortcomings that are not fully appreciated. First, the chronological patterns of cellular and molecular changes in various brain regions remain inadequately defined. Alterations affecting brain response systems beyond the cerebellum remain largely unexplored. Second, the interplay between protein aggregation, cellular stress responses, and neuronal dysfunction requires further clarification across different stages of development. Identifying the critical time windows in which to halt disease onset, rather than merely slowing its progression, is essential. Moreover, the distinct and variable expressivity, along with the age-dependent progression of symptoms in humans, suggests the involvement of diverse pathogenic mechanisms, some of which, such as cognitive impairment in MSS patients, may be inadequately represented in animal models. There is, therefore, an urgent need for comprehensive characterization of existing preclinical models and adjusting the current approaches to study ARCAs and MSS.

In this study, we utilised the woozy mouse model to evaluate a deep phenotyping pipeline spanning from early life (5 weeks of age) to middle adulthood (26 weeks of age). Through the evaluation of cognitive function and motor performance across the lifespan (5–16, and 26 weeks of age), we demonstrate that the analytical pipeline used provides robust information relevant to understanding changes that occur during the pathology development in a mouse model of MSS, thereby better defining the real potential of this preclinical model. Moreover, we investigated the specific sex and hormone contribution in this model. This approach is essential for advancing our understanding of disease mechanisms and developing effective treatments for these devastating neurological disorders.

## Materials and Methods

### Animal Models

*Sil1* homozygous (woozy) mice (*Sil1*^*wz*^) were produced by crossing *Sil1* heterozygous mice (*Sil1*^*ht*^) (CXB5/By-*Sil1*^wz^/J, JAX stock #003777), supplied by The Jackson Laboratory (Maine, USA). Mice were housed with their littermates and aged until weaning (PND 23–24). The genotype of the animals was confirmed using the standard PCR assay (Jackson Laboratories Protocol #22,452) performed on DNA isolated from ear punches [25]. After PCR, the amplified sequences were analysed through 1.5% agarose gel electrophoresis, and the genotype was determined based on the size of the sequences: *Sil1*^*wz*^ = 758 bp; *Sil1*^*ht*^ = 626 bp and 758 bp; wild type = 626 bp.

Mice were housed in groups of four to five per cage (Tecniplast, Buguggiate, Italy) with wood chips as bedding (Caipet, San Severo, Italy). The mice were maintained in a temperature-controlled room on a 12-h light–dark cycle and had free access to food and water. Starting at the 5^th^ week of life, the first batch of animals were weighed weekly and tested at the accelerating rotarod and beam walking on two different days. In the 10th week, a nesting building test was performed the night before the first motor test. At the 14^th^ week, the second time point of the nesting building was performed, together with the pole test, for two consecutive days. At the 16^th^ week, animals performed the second pole and inverted grid tests. The day after the last test, half of the animals for each experimental group were randomly selected and sacrificed with cervical dislocation. The other animals were maintained in the same housing conditions until the 26^th^ week. A second batch of animals was tested at the 5^th^ and 14^th^ weeks of life in the novel object recognition and Y-maze tests on two consecutive days. A total of 44 mice, comprising 22 *Sil1*^*wz*^ and 22 *Sil1*^*ht*^, equally distributed by sex, were used for the characterisation of this model.

All experimental procedures were approved by the CAST animal welfare survey board, on behalf of the Italian Ministry of Health (formal license 596/2021 PR, delivered to M.S.), and followed European Community specifications regarding the use of laboratory animals.

### Estrous Cycle Staging

To establish the regularity of the cycle, evaluate the contribution of sex hormones to the motor tests, and avoid an unbalanced distribution of females within each individual test, estrous cycle tracking was carried out for four entire weeks during the tests. Briefly, a vaginal smear was performed by gently pipetting sterile water inside the vaginal opening, then depositing the sample on a microscope slide. Once dried, the sample was stained with 0.1% crystal violet solution and observed under a light microscope to establish the estrous phase based on cellular composition, as previously described [26]. We attempted to equally distribute female mice across testing days and avoid an excessive number of Proestrus/Estrous (Pro/Est) or Metestrus/Diestrus (Met/Die) females within the same test.

### Motor Assessment

#### Accelerating Rotarod Test

To evaluate sensorimotor deficits [27], the motor functions and neuromuscular coordination were assessed with the accelerating Rotarod 7650 model (Accelerating Model, Ugo Basile, Biological Research Apparatus, Varese, Italy), as previously described [28]. On the day of the test, mice were trained three times, for 60 s at a constant acceleration of 4 rpm before starting. For the test, animals were placed on the rotating bar (constant at 7 rpm) and allowed to acclimatise for 30 s. The acceleration began at 7 rpm and accelerated up to 40 rpm, at a constant rate of 0.11 rpm/s for a maximum of 300 s. Motor coordination and fatigue were evaluated by measuring animals’ latency to fall, recorded as the endpoint measure. Animals performed three times, with a rest of 30 min between each trial, and the average time was used for statistical analysis. Animals were weekly tested from the 5^th^ to the 14^th^ week of life. Instead of representing data as the individual latency time expressed in s, we choose to represent the values as the percentage (%) with respect to the individual maximum time in the apparatus.

#### Beam Walking Test

Mice were trained two days before being tested to walk along a custom-made PVC round bar 0.8 cm wide, 80 cm long, suspended 40 cm above bedding material, with a safe platform at the end [29]. On the day of the test, animals were given three trials, with at least 5 min between each trial. Mice were video recorded during their performance and the time to traverse the beam, the number of hindfoot missteps, and eventual falls during the trials were scored. The average time to travel the beam and reach the platform, together with the number of missteps (considered when the mice slid with their hind limbs along the round beam), scored during the three trials were used for statistical analysis. The video analysis was performed by an operator blinded to the experimental conditions. A cutoff of 60 s was chosen as the maximum time in case the animals fell or spent more time traversing the beam. Animals were tested weekly starting from the 5^th^ to the 12^th^ week of life.

#### Pole Test

A custom-made vertical PVC round pole 0.8 cm wide, 50 cm long, covered with a thin rope twisted on its surface, was positioned on a base and placed into the mouse home cage, as previously described [30]. During the morning of the test, mice were trained to turn themselves from upward on top of the pole and descend the length of the pole to return to their home cage. On the day of testing, animals were given three trials and were video recorded. The time required for the animals to orient themselves facing in a downward direction (T-turn) and to descend the base of the pole (T-total) were scored. The average T-turn and T-total times were used for statistical analysis. A cutoff of 30 s was chosen as the maximum T-turn time, while a cutoff of 60 s was chosen as the maximum T-total time. Animals were tested at their 14^th^ and 16^th^ weeks of life.

#### Inverted Grid Test

The inverted grid test was used to assess grip strength and muscle endurance [31]. Mice were placed onto a suspended stainless-steel wire grid (40 × 40 cm, wires spaced 1 cm apart) located 40 cm above the floor over a soft paper chip bedding. After a mouse was placed at the centre of the wire grid, the apparatus was inverted, and the test began. The trial was videotaped, and latency to drop down from the wire grid (cutoff time 120 s), together with immobility time, time spent in the different zones of the apparatus, and the number of times the animals leave the grid with their forelimbs or hindlimbs were scored and considered for the analysis. An operator blinded to the experimental conditions evaluated and scored all the parameters. Mice were tested two days after the last motor test at their 16^th^ week of life.

### Cognitive Behaviour

#### Nesting Building

Approximately 1 h before the dark phase, mice were individually moved to a testing cage equipped with wood chip bedding and 3 g of paper folded twice and placed in the back part of the cage. The following morning, an operator removed the cage lid and took a picture of the entire cage, and of the opened rectangular piece of paper from above, to evaluate the movement of the paper inside the cage, the degree of fold opening, and the percentage of gnawing by the mouse. Mice were individually caged for their entire dark phase, from 6 p.m. to 9 a.m., at their 10^th^ and 14^th^ week of life. Differently from the original protocol [32] but similarly to previous studies [33], we used the following scoring to evaluate the individual parameter: paper movement—1 to 5 (1 = no movement; 3 = paper moved to the middle of the cage; 5 = paper moved to the end of the cage); degree of opening—1 to 3 (1 = paper closed; 2 = paper half opened; 3 = paper completely opened); gnawing percentage—1 to 5 (1 = not gnawed paper at all; 3 = 20% of paper gnawed; 5 = more than 40% of paper gnawed). Three operators blinded to the experimental conditions evaluated and scored the test parameters, and the average of each parameter was used for statistical analysis.

#### Novel Object Recognition Test

The Novel Object Recognition test (NOR) was performed as previously described [34]. Briefly, two Plexiglas (30 × 15 cm) cages were used for the experiment. During the habituation phase, mice were individually placed for 10 min per day for 2 consecutive days in the empty cages. On day 3, mice were placed in the cage and exposed to two identical plastic objects 15 cm apart and allowed to explore the objects for 8 min before returning to their home cage. Between each trial, objects were thoroughly washed with 70% ethanol and dried to remove any scent residue. On day 4, mice were returned to the experimental cage where one of the two familiar objects was replaced with a differently shaped object (novel object). A 5 min probe test was recorded while mice were allowed to freely explore the familiar and novel objects. NOR performance scores were quantified by monitoring the time spent exploring both familiar and novel objects. Mice's behaviour was considered explorative when the animal heads leaned within 1 cm from the object and vibrissae moved. Proximity, chewing, or standing without overt signs of object exploration were not considered exploratory behaviour. Parameters included in the analysis were: the percentage of time spent with the novel object and the discrimination index (DI). The latter is defined as the fraction of exploration time spent with a novel object, calculated as [(A–B)/(A + B)] × 100 – with A the time spent exploring the novel object and B the time spent exploring the familiar one.

#### Y-maze

Working memory and exploratory activity were measured by employing a Y-maze apparatus as described elsewhere [35]. The apparatus consists of a Y-shaped structure made of black plexiglass walls with dimensions 39.5 × 8.5 × 13 cm. Mice, naive to the maze, were individually placed at the end of one arm of the apparatus and allowed to move freely through the maze. Each 10-min session was recorded, and the number of arm entries and alteration patterns were analysed. The percentage of alternations was calculated as follows: % alternations = (total number of alternations/numbers of arm entries) × 100.

#### Histological Analysis

Immediately after sacrifice, the cerebellum, quadriceps, soleus, and gastrocnemius muscles were embedded in OCT and frozen in liquid nitrogen prior to being stored at −80 °C. Transverse tissue sections, each 10 µm thick, were cut from the middle portion of the quadriceps, gastrocnemius, and soleus muscles using a cryostat (CM1950; Leica, Wetzlar, Germany) at − 25° C. The sections were then mounted onto glass slides (six sections per slide) and visualised using the haematoxylin and eosin (H&E) staining. All stained sections were scanned (20 × objective) to measure the muscular fibre area (cross-sectional area – CSA) using freeware Image-J software, version 1.54 g (National Institutes of Health, USA). This analysis was performed using seven randomly selected regions of interest in 5–6 cross sections per animal.

### Molecular Analysis

#### Protein Analysis

A total of 3 *Sil1*^wz^ and 3 *Sil1*^*ht*^, equally distributed in sex, were randomly selected for the molecular analysis. Two days after the last motor assessment test, mice were sacrificed by cervical dislocation, and tissues were quickly frozen and stored at − 80 °C until molecular assays. Frozen mouse gastrocnemius and soleus muscles (~ 100 mg) were homogenised in ice-cold lysis buffer (0.5 M Tris–HCl [pH 7.4], 1.5 M NaCl, 2.5% deoxycholic acid, 10% NP-40, 10 mM EDTA) using ultrasound (10 × pulse, 0.5 amplitude, 50% efficiency), prior to being centrifuged at 12,000 × g for 20 min at 4 °C [36–38]. The concentration of the protein supernatant was determined using the Bradford assay [39]. The proteins were mixed with Laemmli buffer (31.5 mM Tris–HCl [pH 6.8], 10% glycerol, 1% SDS, 0.005% bromophenol blue), denatured for 10 min at 95 °C, and the same amount of protein (25 µg per well) was separated by SDS-PAGE in a 12.5% gel. After the run, the gel was transferred to a nitrocellulose membrane using a wet/tank blotting system (Bio-Rad, Hercules, CA, USA). Consequently, the membrane was blocked using TBSt (TBS + 1% Tween 20) + 5% milk/BSA (based on the Ab used) for 1 h at RT and was then incubated with antibodies specific for BiP, Pdi, pEIF2α, Chop, Rab11, LC3, and Ckb overnight at 4 °C. After primary antibody incubation, the membrane was washed × 3 in TBSt (0.1%) and incubated with H&L chain specific peroxidase-conjugate secondary anti-rabbit (#401,315, Calbiochem) or anti-mouse (#401,215, Merck Millipore) antibody for 1 h at RT before washing × 3 with TBSt (0.1%). Antibody binding was detected using the Alliance Atom (UVITEC, Cambridge, UK). GAPDH protein abundance was used as a reference control. Data are expressed relative to the GAPDH levels. All the details regarding the antibodies used in the study are reported in Supplementary Table 1.

#### Statistical Analysis

All data are expressed as the mean ± SEM and the statistical analysis was performed with GraphPad Prism 10 software. The multiple t-test, corrected with the Holm-Sidak method, was used to compare data obtained from the locomotor tests performed between the two groups. The unpaired Mann–Whitney t-test was used to analyse the individual parameters not dependent/influenced by other parameters of the same test (e.g., the total latency and the immobility time of the inverted grid test). Unpaired t-test was used to analyse data from the molecular analysis. Spearman’s correlation analysis was used to measure the strength and direction of the relationship between the individual parameters observed in the motor and cognitive tests. The value of p < 0.05 was predetermined as the threshold for statistical significance.

We decided to exclude one male of the *Sil1*^*ht*^ group from all the analysis due to continuously failing the pole test, thus reporting the same comparison for all the performed tests.

## Results

### The Estrous Cycle does not Affect the Motor Behaviour of the Woozy Mouse

We tested the possible impact of sex hormone fluctuation on the locomotor and cognitive (nesting building) tests tracing estrous cycle phases in females through vaginal smear. Upon examining the motor test results of individual mice over the course of several weeks, we observed no differences between Proestrus/Estrus and Metestrus/Diestrus females for the accelerating rotarod, beam walking, pole, inverted grid, and nesting building tests. Therefore, we chose not to subdivide the group of female animals further, as the balance between the Pro/Est and Met/Die phases remained consistent throughout the study Supplementary Fig. 1b. A representative picture of the individual estrous cycle phases, determined through vaginal smear, is reported in Supplementary Fig. 1a.

### Evaluation of the Motor Performances in Woozy Mice

#### Male Woozy Mice Perform Worse than Females and Heterozygous Controls in the Accelerating Rotarod Test

**Fig. 1:**
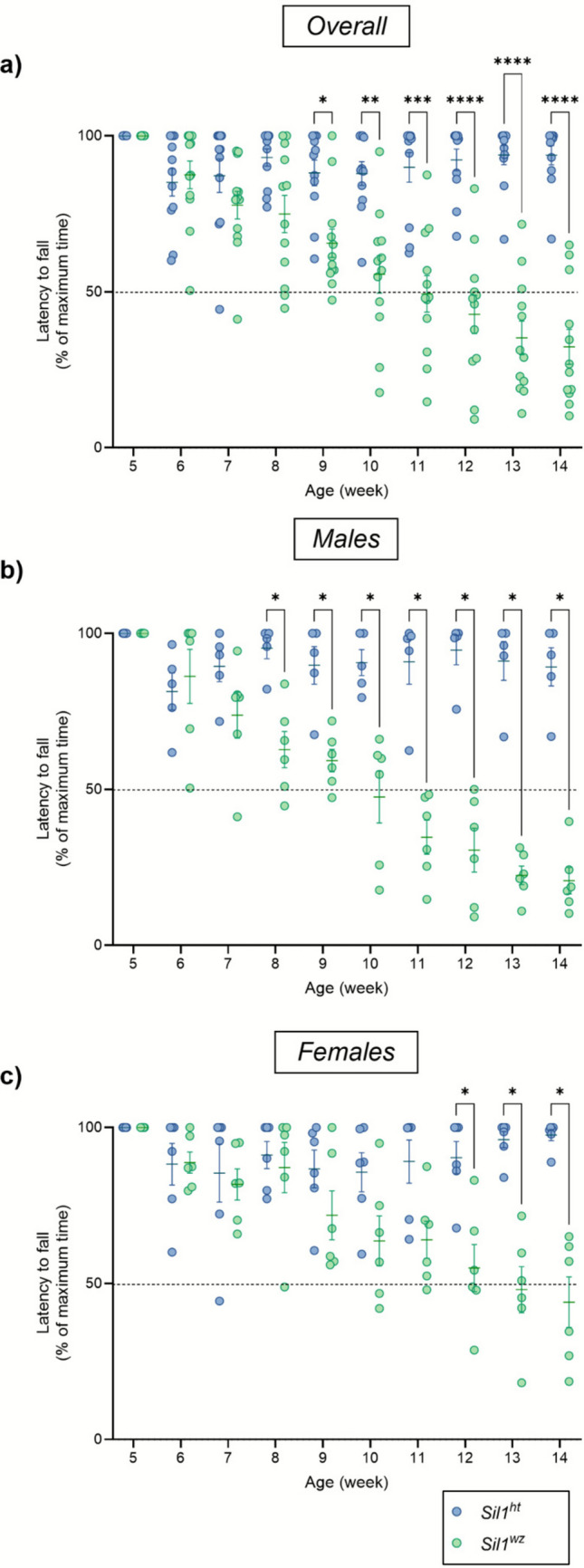
Progression of cerebellar damage shown over the weeks. Latency to fall in the overall population (**a**), male (**b**), female (**c**), *Sil1*^*ht*^ (**d**), and *Sil1*^*wz*^ (**e**) mice. Data are represented as scattered dot plots (mean ± SEM, of each group) and expressed as the percentage of the maximum time for the individual animal. *Sil1*^*wz*^ = green circles; *Sil1*^*ht*^ = blue circles. Significant differences are indicated (*p < 0.05, **p < 0.01, ***p < 0.005, ****p < 0.001)

Using the accelerating rotarod test, it is possible to evaluate mice's motor functions and neuromuscular coordination, as indicated by a reduction in latency time [27, 28]. The *Sil1*^*wz*^ mice exhibited a significant reduction in latency time compared to the control group starting on the 9^th^ week (*Sil1*^wz^: 65.55 ± 4.54%, *Sil1*^*ht*^: 88.09 ± 4.11%; *p* = 0.0182) (Fig. 1a). The decrease in latency time continued until the 14^th^ week, with the *Sil1*^wz^ group reaching one-third of their maximum time. We then examined sex-specific contributions in the differences between the two groups. In males, a significant reduction in latency time was observed in *Sil1*^wz^ mice compared to *Sil1*^*ht*^ mice starting at the 8^th^ week, and this difference persisted throughout the motor test until the 14^th^ week (Fig. 1b). In females, the decrease in latency time observed in the *Sil1*^*wz*^ group began at the 9^th^ week, though it reached statistical significance only at the 12^th^ week (Fig. 1c). Given the reported sex-based variability in disease onset, we also directly compared males and females within the *Sil1*^*wz*^ and *Sil1*^*ht*^ groups. As shown in Supplementary Fig. 2a and b, *Sil1*^*ht*^ and *Sil1*^wz^ females show better motor performances compared to *Sil1*^*ht*^ and *Sil1*^*wz*^ males, respectively, although this effect did not reach statistical significance in most cases (Supplementary Fig. 2b). The weekly average times and corresponding p-values for each individual week are reported in Supplementary Table 2.

#### Male Woozy Mice Perform Worse than Both Female and Heterozygous Controls in the Beam Walking Test

**Fig. 2:**
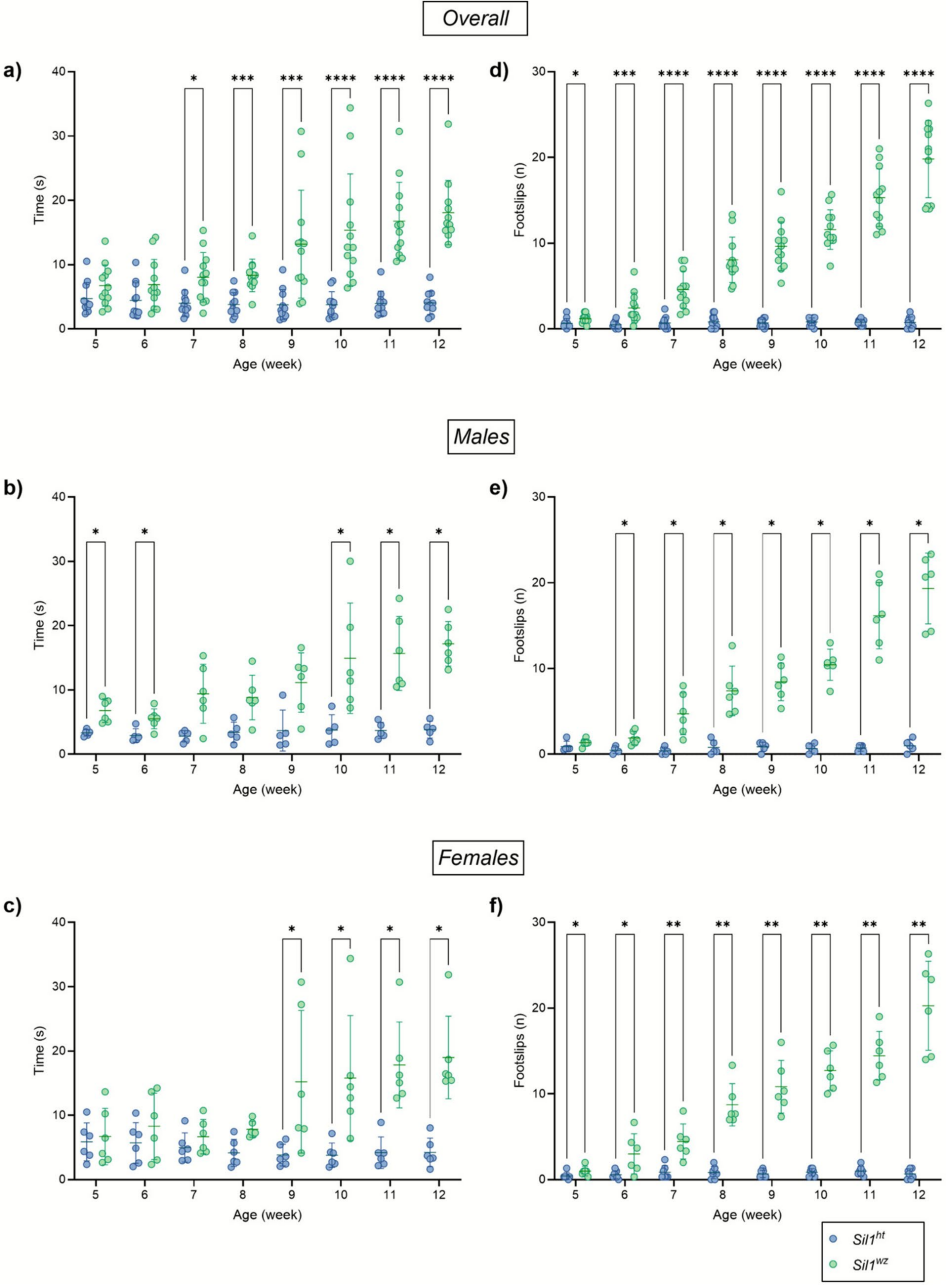
The beam walking test highlights the coordination problems manifested by the *Sil1*^*wz*^ mice. Time to traverse the bar and number of contralateral falls in the overall population (**a**, **d**), male (**b**, **e**), and female (**c**, **f**) mice. Data are represented as scattered dot plots (mean ± SEM, of each group). *Sil1*^*wz*^ = green circles; *Sil1*^*ht*^ = blue circles. Significant differences are indicated (*p < 0.05, **p < 0.01, ***p < 0.005, ****p < 0.001)

The beam walking test effectively assesses fine motor coordination and balance. Animals that take longer to traverse the beam and exhibit an increased number of contralateral falls demonstrate reduced balance and coordination. Starting from the 7^th^ week of age, *Sil1*^*wz*^ mice exhibited a significantly longer time to travel the beam compared to *Sil1*^*ht*^ mice *(Sil1*^*wz*^: 8.03 ± 1.11 s, *Sil1*^*ht*^: 4.00 ± 0.62 s; *p* = 0.0208), which continued to increase every week until the end of the test (Fig. 2a). Stratifying the data by sex revealed a clear distinction between male and female subjects. While *Sil1*^*wz*^ males showed a significantly higher time to travel the beam compared to the *Sil1*^*ht*^ group starting from the 5^th^ week (Fig. 2b), *Sil1*^*wz*^ females began to exhibit significant differences relative to their counterparts starting at the 9^th^ week (Fig. 2c). An analysis of contralateral foot slips during beam traversal revealed a significant increase in the overall population starting from the first week of testing (*Sil1*^*wz*^: 1.18 ± 0.15, *Sil1*^*ht*^: 0.65 ± 0.17; *p* = 0.0150) (Fig. 2d). When analysing males and females separately, a significant increase in contralateral foot slips was observed from the second week in males and from the first week in females, (Fig. 2e and f). When comparing males and females within the same genotype, no significant differences were observed between the groups (Supplementary Fig. 3a-d). The weekly average beam traversal time, number of foot slips, and corresponding p-values for each individual week are provided in Supplementary Table 3.

#### Male Woozy Mice Perform Worse than Both Female and Heterozygous Controls in the Pole Test

**Fig. 3:**
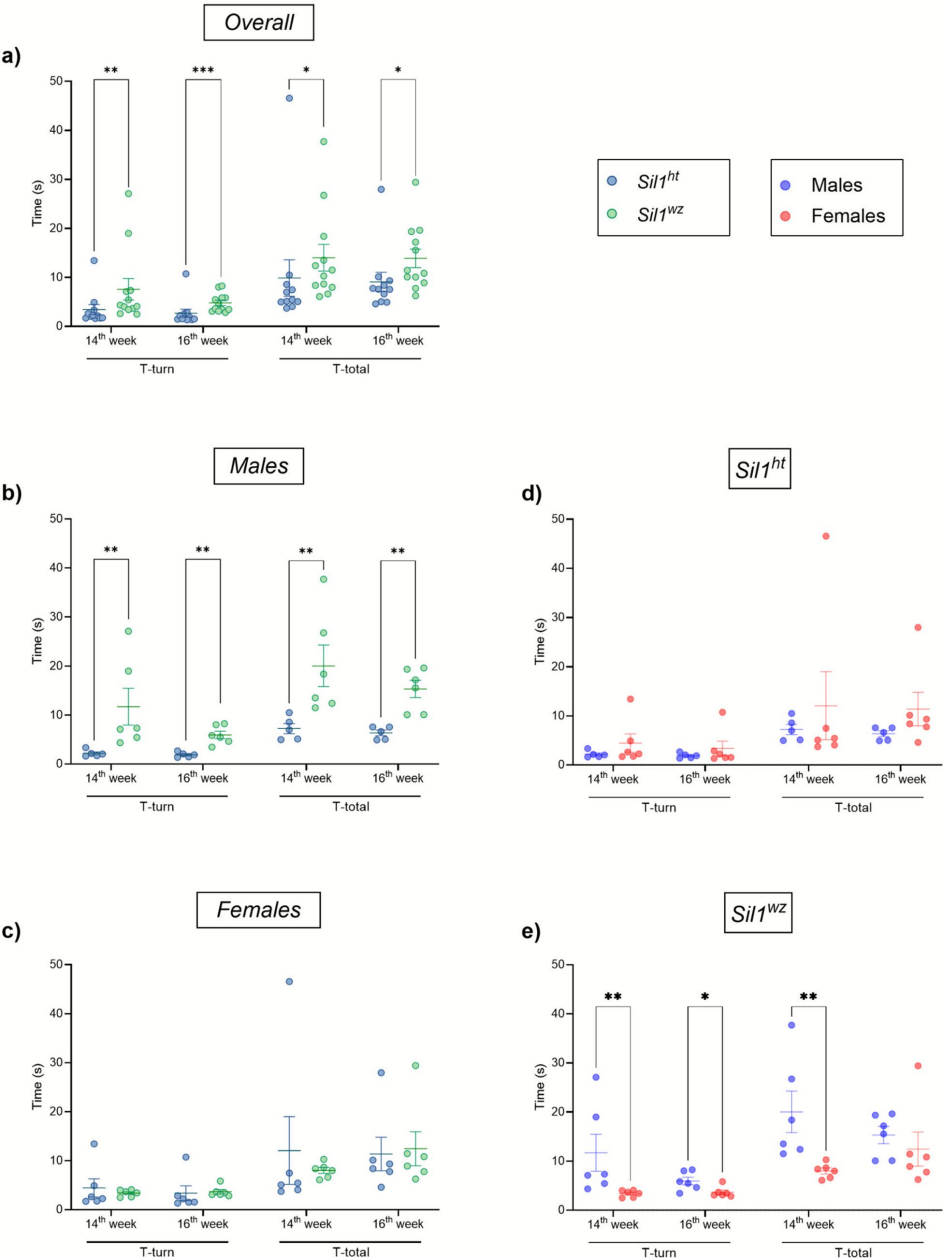
Genotype- and sex-dependent impairments in the pole test manifested by the *Sil1*^*wz*^ mice. Turning (T-turn) and total (T-total) time at the 14^th^ and 16^th^ week of life in the overall population (**a**), male (**b**), female (**c**), *Sil1*^*ht*^ (**d**), and *Sil1*^*wz*^ (**e**) mice. Data are represented as scattered dot plots (mean ± SEM, of each group). *Sil1*^*wz*^ = green circles; *Sil1*^*ht*^ = blue circles; Males = blue circles; Females = red circles. Significant differences are indicated (*p < 0.05, **p < 0.01, ***p < 0.005)

In this test, the animals often orient themselves downwards and descend the length of the pole to return to their home cage. Prolonged turn (T-turn) and total (T-total) times indicate cerebellar damage, which leads to impaired coordination. In line with the results of the previously performed motor tests, *Sil1*^*wz*^ mice showed a significant increase in the time of turning (T-turn) once attached to the pole with respect to the controls, both at the 14^th^ (*Sil1*^*wz*^: 7.55 ± 2.19 s, *Sil1*^*ht*^: 3.42 ± 1.05 s; *p* = 0.0045) and at the 16^th^ week (*Sil1*^*wz*^: 4.81 ± 0.55 s, *Sil1*^*ht*^: 2.70 ± 0.82 s; *p* = 0.0009) (Fig. 3a). When considering the total time (T-total), defined as the time taken to descend the pole after fully turning from the top, a significant increase in descent time was observed in the *Sil1*^*wz*^ group, at the 14^th^ (*Sil1*^*wz*^: 14.02 ± 2.72 s, *Sil1*^*ht*^: 9.87 ± 3.72 s; *p* = 0.0221) and the 16^th^ week (*Sil1*^*wz*^: 13.88 ± 1.91 s, *Sil1*^*ht*^: 9.09 ± 1.96 s; *p* = 0.0221) (Fig. 3a). This test revealed a much clearer distinction between the two sexes compared to the results observed in the Accelerating Rotarod and Beam Walking tests. Specifically, *Sil1*^*wz*^ males required more time than *Sil1*^*ht*^ to complete both the T-turn and T-total at each time point (Fig. 3b); one male of the *Sil1*^*ht*^ group was excluded from the analysis due to continuously failing the test. In females, no statistically significant differences were observed between the two groups for the parameters and time points considered (Fig. 3c). When comparing sexes within the same genotype, no differences were observed in *Sil1*^*ht*^ animals (Fig. 3d). However, significant differences were observed between male and female *Sil1*^*wz*^ mice (Fig. 3e). Specifically, *Sil1*^*wz*^ females show a better performance in the pole test with respect to *Sil1*^*wz*^ males for the T-turn at both weeks and for the T-total at the 14^th^ week of life. All group means and p-values are reported in Supplementary Table 4.

#### Woozy Mice Exhibit Similar Latency Times but Move Less than Heterozygous Controls in the Inverted Screen Test

**Fig. 4:**
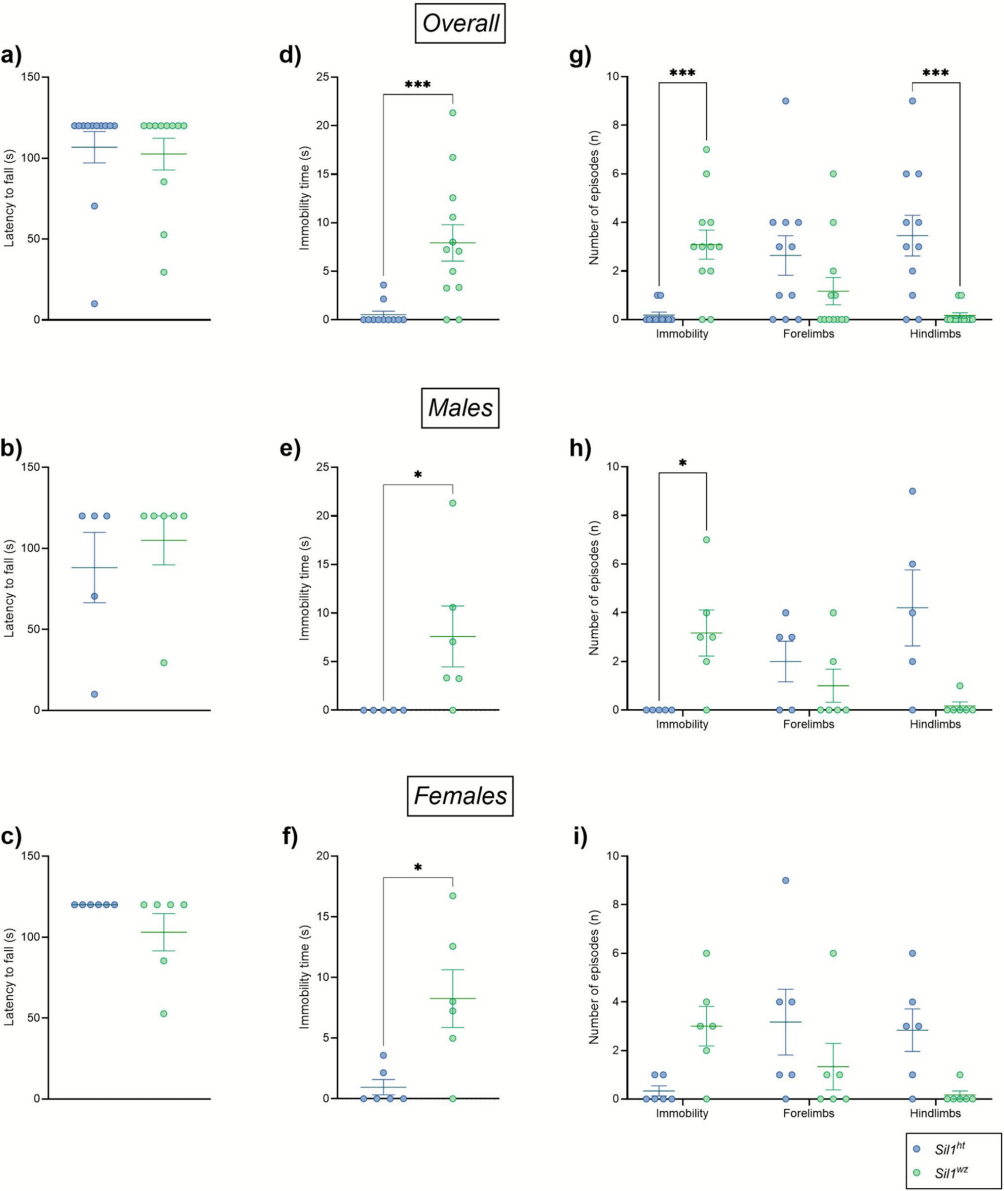
Muscular coordination impairment highlighted by the increased immobility in the *Sil1*^*wz*^ mice. Latency to fall, number of episodes, and immobility time for the individual parameter scored in the overall population (**a**, **d**, **g**), male (**b**, **e**, **h**), and female (**c**, **f**, **i**) mice. Data are represented as scattered dot plots (mean ± SEM, of each group). *Sil1*^*wz*^ = green circles; *Sil1*^*ht*^ = blue circles. Significant differences are indicated (*p < 0.05, ***p < 0.005)

The recorded latency to fall in this test measures grip strength and muscle endurance. No differences in the latency time to drop from the grid were observed between the two groups (*Sil1*^*wz*^: 102.5 ± 9.80 s, *Sil1*^*ht*^: 106.7 ± 9.70 s; *p* = 0.6476) (Fig. 4a, b, c). Although no differences in latency time were observed between the *Sil1*^*wz*^ and *Sil1*^*ht*^ groups, the number of episodes (n) in which the animals stopped moving was significantly higher in the *Sil1*^*wz*^ group compared to the *Sil1*^*ht*^ group (*Sil1*^*wz*^: 3.08 ± 0.60, *Sil1*^*ht*^: 0.18 ± 0.12; *p* = 0.0007). This effect remained evident after sex-based stratification, although it did not reach statistical significance in females (Fig. 4d, e, f). In addition to the total number of episodes (n), the *Sil1*^*wz*^ mice also showed an increase in the total time (s) spent without movement. A significant difference between the two groups was observed in the overall population (*Sil1*^*wz*^: 7.92 ± 1.88 s, *Sil1*^*ht*^: 0.52 ± 0.36 s; *p* = 0.0005) (Fig. 4g), in males (*Sil1*^*wz*^: 7.59 ± 3.12 s, *Sil1*^*ht*^: 0.00 ± 0.00 s; *p* = 0.0152), and in females (*Sil1*^*wz*^: 8.26 ± 2.38 s, *Sil1*^*ht*^: 0.95 ± 0.63 s; *p* = 0.0216) (Fig. 4h and i). However, when stratifying the data by sexes within the same genotype, no differences were observed for any of the parameters analysed (Supplementary Fig. 4a-d). Interestingly, when analysing the number of episodes in which animals leave the grid with their hindlimbs but not the forelimbs, a significant increase was observed in the *Sil1*^*ht*^ group compared to the *Sil1*^*wz*^ (*Sil1*^*wz*^: 0.17 ± 0.11, *Sil1*^*ht*^: 3.46 ± 0.84; *p* = 0.0009) (Fig. 4d). However, this difference was no longer statistically significant after sex-based stratification (Fig. 4e, f). Detailed results and p-values are reported in Supplementary Table 5.

### Evaluation of the Cognitive Behaviour in Woozy Mice

#### Woozy Mice Performed Worse than Heterozygous Controls in the Nesting Building Test

**Fig. 5:**
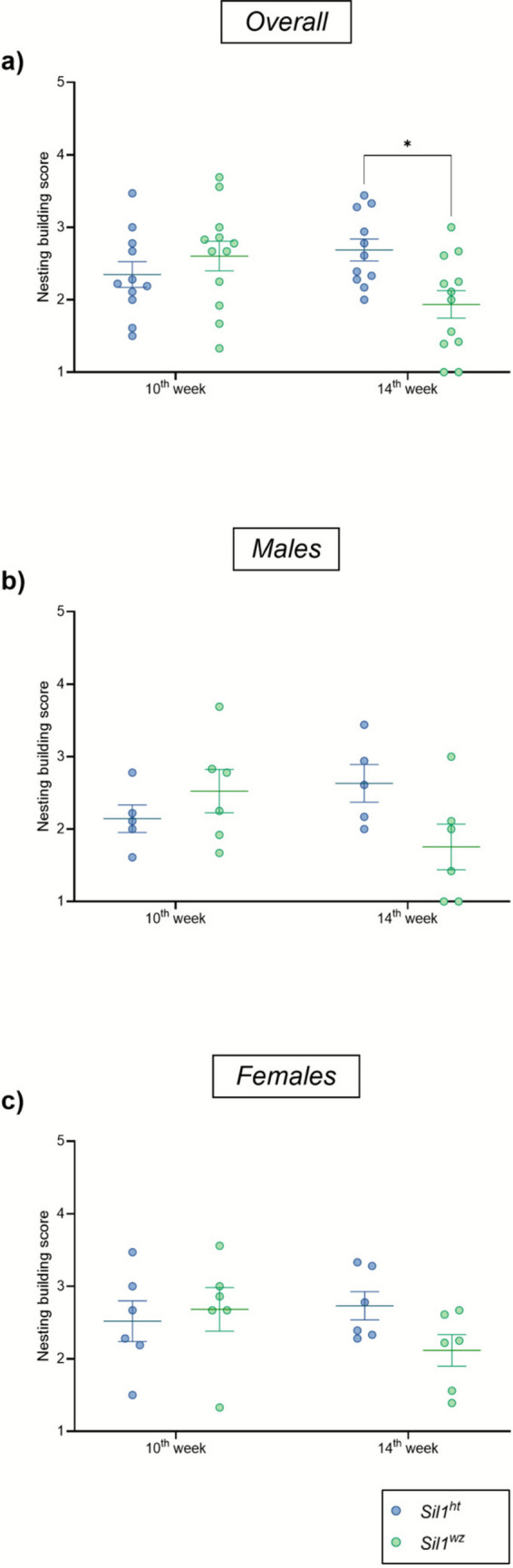
Impaired nesting building behaviour in the *Sil1*^*wz*^ mice. Average score of the paper movement, degree of opening, and gnawing percentage at the 10^th^ and 14^th^ week of life in the overall population (**a**), male (**b**), and female (**c**) mice. Data are represented as scattered dot plots (mean ± SEM, of each group). *Sil1*^*wz*^ = green circles; *Sil1*^*ht*^ = blue circles. Significant differences are indicated (*p < 0.05)

Building the nest is a spontaneous behaviour in mice that, when diminished, serves as an indirect measure of impaired cognitive function and decreased neuropsychiatric health. In the nesting building assay, no significant difference was observed between the *Sil1*^*wz*^ and *Sil1*^*ht*^ groups at the 10^th^ week (*Sil1*^*wz*^: 2.60 ± 0.20, *Sil1*^*ht*^: 2.55 ± 0.18; *p* = 0.3084). By the 14^th^ week, however, the *Sil1*^*wz*^ group had a much lower total score than the *Sil1*^*ht*^ group (*Sil1*^*wz*^: 1.94 ± 0.19, *Sil1*^*ht*^: 2.69 ± 0.15; *p* = 0.0199) (Fig. 5a). Specifically, a decrease was observed in all three parameters compared to the *Sil1*^*ht*^ mice. However, the third parameter (gnawing percentage) was the primary contributor to the difference observed in the total score at the 14^th^ week (Supplementary Fig. 5 and Supplementary Table 6). No specific contribution was observed based on the mice's sex (Fig. 5b and c), nor were there differences when comparing the sexes within the same genotype, also considering the individual parameters (Supplementary Fig. 5d and e).

#### Woozy Mice Performed Similarly to Heterozygous Controls in Novel Object Recognition And Y-maze Tests

Spatial working memory and learning were evaluated through the NOR and Y-maze tests. However, in the NOR test, no differences were observed between the *Sil1*^*wz*^ and *Sil1*^*ht*^ groups at the 5^th^ week (*Sil1*^*wz*^: 59.08 ± 7.06, *Sil1*^*ht*^: 66.03 ± 5.02; *p* = 0.7780) and at the 14^th^ (*Sil1*^*wz*^: 62.53 ± 8.07, *Sil1*^*ht*^: 66.48 ± 7.67; *p* = 0.7780) in the time spent approaching the novel object, as measured by the discrimination index (%) (Supplementary Fig. 6a). No differences were observed after stratifying the data by sex or genotype (Supplementary Fig. 7b-e). All group’s means and p-values are reported in Supplementary Table 7. In the Y-maze test, no significant differences were observed in arm alternation between the *Sil1*^*wz*^ and *Sil1*^*ht*^ groups, either at the 5^th^ week (*Sil1*^*wz*^: 69.60 ± 4.20, *Sil1*^*ht*^: 68.00 ± 2.62; *p* = 0.9639) or at the 14^th^ week of life (*Sil1*^*wz*^: 67.30 ± 2.93, *Sil1*^*ht*^: 66.00 ± 3.06; *p* = 0.9639) (Supplementary Fig. 7a). Consistent with the findings from the NOR test, no significant differences between groups were observed in the Y-maze when the data were stratified by sex (Supplementary Fig. 7b and c) or genotype (Supplementary Fig. 7d and e). All group means, and p-values are reported in Supplementary Table 8.

#### Strong Inter-correlations among the Individual Motor Test Outcomes

**Fig. 6:**
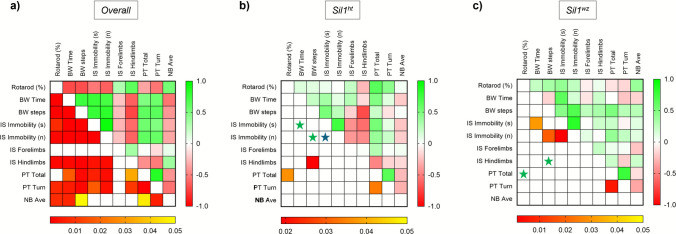
Genotype-specific correlation trend in the motor and cognitive tests. Spearman’s correlation analysis between the individual values of motor (Rotarod, Beam walking, Inverted screen, Pole) and cognitive (Nesting) tests considering the overall population (**a**), *Sil1*^*ht*^ (**b**), and *Sil1*^*wz*^ (**c**) mice. Cells filled in green to red gradient of the heat maps (upper part) represent Spearman’s r; cells filled in yellow to red gradient (lower part) represent p values (empty cells stand for p values greater than 0.05). The stars show the missed correlation between two parameters in the same test (blue) or in different tests (green) comparing the two experimental groups. BW = Beam Walking; IS = Inverted screen test; PT = Pole test; NB = Nesting building test

Spearman’s correlation analysis of individual mouse performance across the various motor and cognitive tests revealed strong correlations among all the tests conducted (Fig. 6a). When stratifying the mice population based on their genotype, we observed a different trend between *Sil1*^*wz*^ and *Sil1*^*ht*^ mice. The *Sil1*^*ht*^ group exhibited a strong inverse correlation between rotarod latency to fall and total time in the pole test, as well as between contralateral falls in the beam walking test and hindlimb performance on the inverted screen test (Fig. 6b). In the *Sil1*^*wz*^ group, a strong positive correlation was observed between inverted screen immobility time and the number of immobility episodes, as well as with beam walking time and contralateral falls (Fig. 6c). All parameters included in the correlation analysis were derived from the final week of each individual test. (rotarod week 14, beam walking week 12, inverted screen week 16, pole test week 14, nesting building week 14). Group means and p-values are reported in Supplementary Table 9.

#### Affected Muscles in Woozy Mice Exhibited Reduced Cross-sectional Area and Upregulation of Stress Markers

**Fig. 7:**
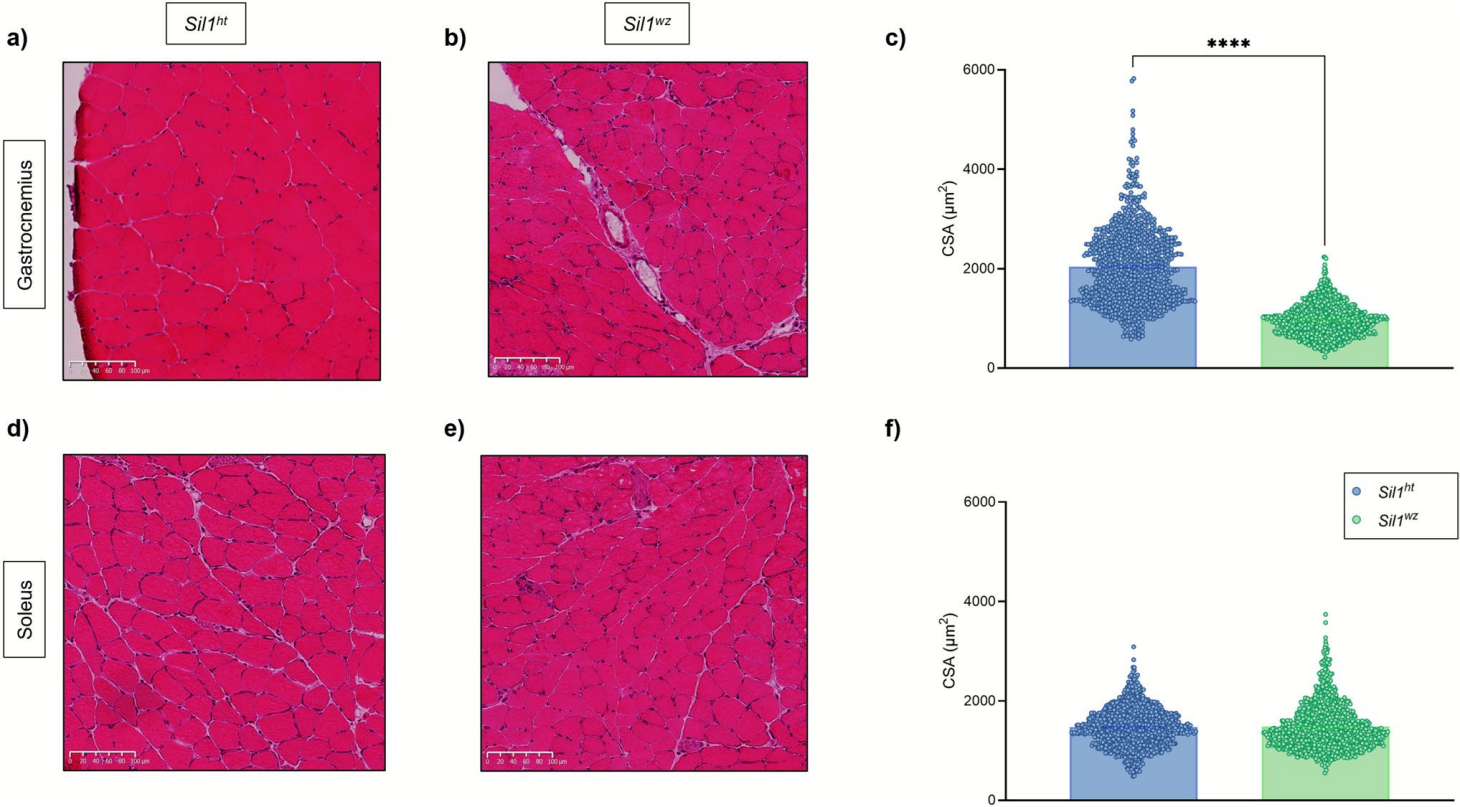
Cross sectional area differences in the glycolytic but not in the oxidative muscles between the *Sil1*^*wz*^ and *Sil1*^*ht*^ mice. Hematoxylin & Eosin staining of gastrocnemius (**a** and **b**) and soleus (**d** and **e**) cross sections. Cross-sectional area (CSA—µm^2^) of gastrocnemius (**c**) and soleus (**f**) muscular fibres measured in 26-week-old *Sil1*^*ht*^ and *Sil1*^*wz*^ mice. Data are represented as scattered dot plots (mean ± SEM, of each group). *Sil1*^*wz*^ = green circles; *Sil1*^*ht*^ = blue circles. Significant differences are indicated (****p < 0.001). Scale bar corresponds to 100 µm

Measurement of the gastrocnemius CSA in 26-week-old mice revealed a statistically significant reduction in muscle fibre CSA in *Sil1*^*wz*^ mice compared to *Sil1*^*ht*^ mice (*Sil1*^*wz*^: 974.3 ± 9.95, *Sil1*^*ht*^: 2037 ± 23.66; p < 0.0001) (Fig. 7c). While a clear difference between the two groups was observed in the gastrocnemius muscle (Fig. 7a, b and c), no significant differences were evident in the soleus muscle (Fig. 7d, e and f) (*Sil1*^*wz*^: 1486 ± 14.31, *Sil1*^*ht*^: 1473 ± 12.05; *p* = 0.1145). Finally, no significant sex-based differences were observed, differently from what observed considering the overall population, in the CSA of either the gastrocnemius or soleus muscles (Supplementary Fig. 8).

**Fig. 8:**
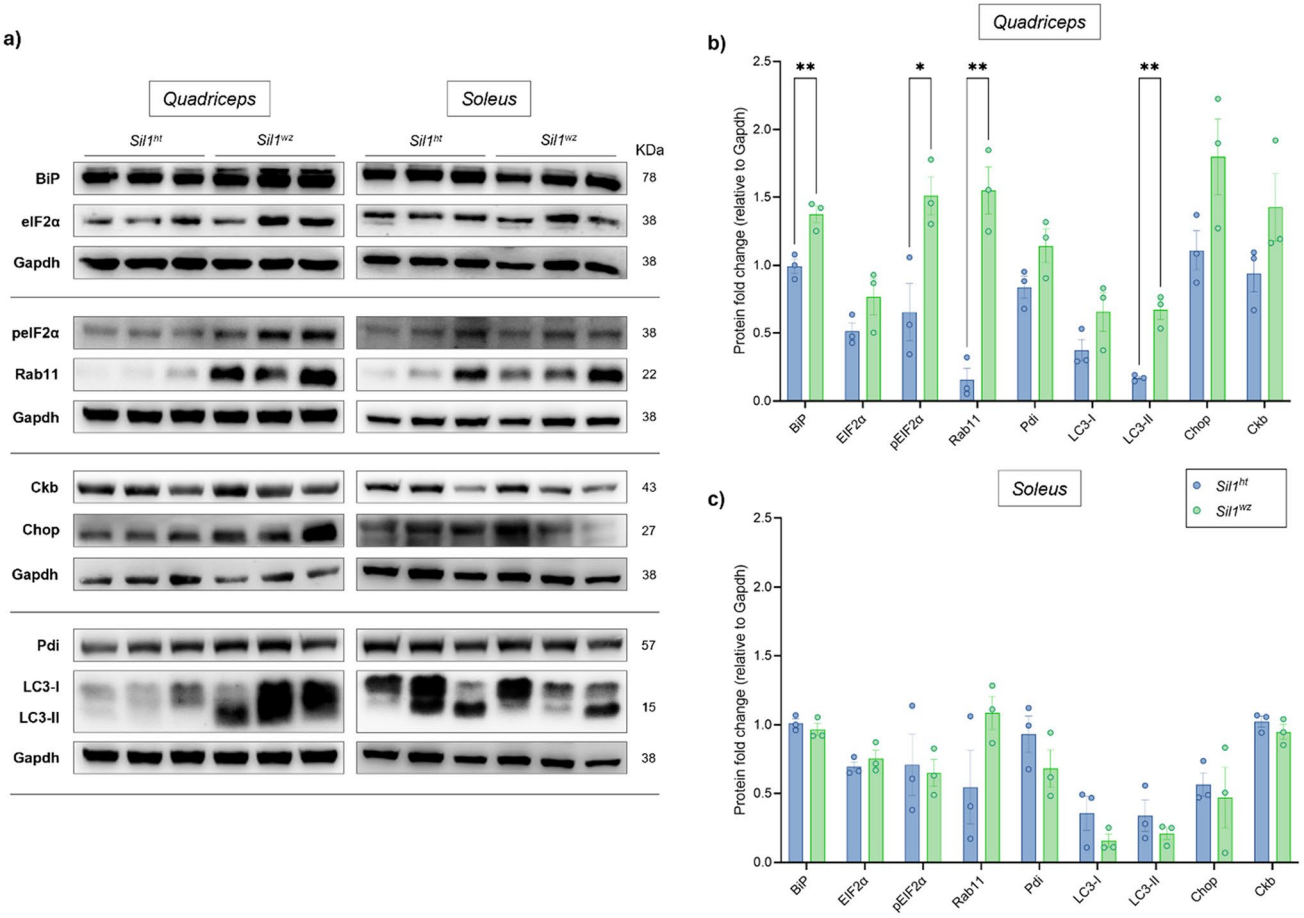
Increased levels of proteins associated with the unfolded protein response and involved in the proteolysis process in the *Sil1*^*wz*^ mice. Western Blot analysis performed in 26-week-old *Sil1*^*ht*^ and *Sil1*^*wz*^ mice (**a**). Protein fold change (relative to Gapdh) observed in the quadriceps (**b**) and soleus (**c**) of *Sil1*^*ht*^ and *Sil1*^*wz*^ mice. Significant differences are indicated (*p < 0.005, **p < 0.01)

Western blot analysis in the quadriceps of 26-week-old *Sil1*^*wz*^ mice revealed increased protein levels of BiP (*Sil1*^*wz*^: 1.37 ± 0.06, *Sil1*^*ht*^: 0.99 ± 0.05; p = 0.0093), pEIF2α (*Sil1*^*wz*^: 1.51 ± 0.14, *Sil1*^*ht*^: 0.65 ± 0.21; *p* = 0.0279), Rab11 (*Sil1*^*wz*^: 1.55 ± 0.17, *Sil1*^*ht*^: 0.16 ± 0.08; *p* = 0.0019), and the lipidated (LC3-II) form of LC3 compared to *Sil1*^*ht*^ mice (LC3-II: *Sil1*^*wz*^: 0.67 ± 0.07, *Sil1*^*ht*^: 0.17 ± 0.01; *p* = 0.0022) (Fig. 8a and b, and Supplementary Table 10). In the soleus of the same mice, we observed a tendency toward increased Rab11 levels (*Sil1*^*wz*^: 1.09 ± 0.12, *Sil1*^*ht*^: 0.55 ± 0.27), together with decreased Pdi (*Sil1*^*wz*^: 0.68 ± 0.14, *Sil1*^*ht*^: 0.93 ± 0.13) and both forms of LC3 (LC3-I: *Sil1*^*wz*^: 0.16 ± 0.05, *Sil1*^*ht*^: 0.36 ± 0.12; LC3-II: *Sil1*^*wz*^: 0.21 ± 0.04, *Sil1*^*ht*^: 0.34 ± 0.11) levels (Fig. 8a and c, and Supplementary Table 10). However, no statistical significance was reached between the two experimental groups in the soleus (see Supplementary Table 10).

## Discussion

In the present study, we conducted a comprehensive phenotypical and molecular characterisation of the woozy mouse, a preclinical model for cerebellar ataxia and muscular atrophy. Starting with the locomotor assessment, we confirmed findings previously reported in the literature. The accelerating rotarod test highlighted the cerebellar damage in the *Sil1*^*wz*^ beginning from the 9^th^ week of age, in which the latency to fall decreased significantly with respect to the *Sil1*^*ht*^ group. Two prior studies reported analogous outcomes in the same test, with *Sil1*^*wz*^ mice exhibiting significant differences commencing at their 8^th^ [19] and 11^th^ [22] weeks of life.

Hayashi and colleagues used a different protocol in the accelerating rotarod test, using a constant speed of 11 rpm with a 180 s cutoff time instead of a 300 s cutoff time with a continuous acceleration up to 40 rpm [22]. This discrepancy may explain the delayed difference observed in latency time compared to our findings. Unlike previous studies, our work provides a thorough assessment of sex-related differences. Overall, we found that males perform worse in this motor test. Notably, female *Sil1*^*wz*^ mice exhibited a significant reduction in the latency time compared to their respective controls only from the 12^th^ week of age. This sex-based difference is even more evident when considering animals of the same genotype, both in *Sil1*^*wz*^ and *Sil1*^*ht*^, probably due to the massive weight increase observed in males with respect to females during the first weeks of life. Although this result may suggest reduced physical endurance in the animals, performance on the rotarod primarily assesses motor coordination rather than muscle strength [40]. As the rotarod test is only an initial screen for neuromuscular impairment, more complex tests, such as gait analyses, are required to define the motor response further [41].

Next, we considered testing the animals using the beam walking test, one of the most sensitive methods for detecting balance and motor skill deficits [42–44]. This test has been widely used to characterise motor phenotypes in preclinical models of neurodegenerative disorders like Parkinson’s disease [45] and Huntington’s disease [46], as well as in transgenic and knockout animals [29]. A significant increase in the time traversing the beam and the number of contralateral falls was observed in the *Sil1*^*wz*^ mice starting from the first week of the test, similarly to what was previously reported [19]. More specifically, this difference is immediately evident in males across both parameters, whereas in females, the delay in beam traversal time becomes apparent only four weeks later compared to males. Rather than reflecting superior performance by female *Sil1*^*wz*^ mice, this delay likely stems from greater variability among *Sil1*^*ht*^ females, who may not perform as consistently well as *Sil1*^*ht*^ males during the early weeks of testing. However, this test measures muscle coordination independently from sex contribution, and, supporting this interpretation, we did not observe differences between males and females within the same genotype.

The pole test was the third motor assessment used to characterise this animal model. The test proved useful in evaluating various types of neuronal dysfunction that could affect performance in this task. As a result, it has become a valuable tool for assessing the effects of anti-Parkinsonian drugs, among other applications [47–50]. The pole test further confirmed cerebellar dysfunction in *Sil1*^*wz*^ mice, with significantly increased turn time and total time to descend the pole as early as the 14^th^ week of life, coinciding with extensive neocerebellar PC degeneration [16, 19, 22], that became more pronounced by the 16^th^ week of life. This finding is in line with the critical role played by PCs in regulating motor coordination [51]. In agreement, at 16 weeks of age, the differences between the two groups are even more pronounced. In contrast to the results observed in the accelerating rotarod and beam walking tests, here the differences between the two groups are more nuanced when examining female animals alone. In this case, unlike in the beam walking test, the lack of statistical significance is primarily due to the better performance of female *Sil1*^*wz*^ mice compared to male *Sil1*^*wz*^ mice, rather than the variability observed in female *Sil1*^*ht*^ mice, and this claim is further supported when observing the improved performance of female *Sil1*^*wz*^ mice compared to their male counterparts. Consistent with previous reports indicating that wild-type males may exhibit shorter pole descent times than females [52, 53], *Sil1*^*ht*^ males performed slightly better, although not significantly, than females. In contrast, *Sil1*^*wz*^ mice exhibited the opposite pattern. One possible explanation for this phenomenon is the greater increase in body weight observed in *Sil1*^*wz*^ males during the first weeks of life, which is not seen to the same extent in *Sil1*^*wz*^ females during the same period (Supplementary Fig. 9). The combination of weight gain and myofibre degeneration may contribute to the increased pole descent time observed in males. However, these interpretations remain speculative based on the available data.

The final test used to assess muscular coordination and strength was the inverted grid, administered when the mice were 16 weeks old. This test, also referred to as the traction test, is commonly used to assess motor-related deficits in preclinical models of motor disorders [54]. Assuming that the *Sil1*^*wz*^ and *Sil1*^*Gt*^ models are similar and considering that the inverted grid test revealed differences only in 24-week-old animals [23], it was not surprising that no significant differences in latency to fall were observed between the two groups at 16 weeks of age. However, the two experimental groups displayed notable differences in the duration of immobility and in the frequency with which they removed their hindlimbs from the grid. These two aspects highlight the muscular deficits in these mice, primarily due to their alteration of the typical climbing behaviour in inbred mice. We speculate that the total time spent before releasing the grid can be envisioned as an indicator of muscular strength, whereas the duration of immobility and the frequency of hindlimb detachment reflect not only physical endurance but also the extent of cerebellar damage. In a study by Tillerson and Miller, forepaw faults observed while the animal is on the grid were shown to accurately reflect striatal dopaminergic levels in the 1-methyl-4-phenyl-1,2,3,6-tetrahydropyridine (MPTP)-treated mouse model of Parkinson’s disease [31]. A forepaw fault was considered when an animal attempted to move or place its forepaw during a weight-shifting (place-shifting) movement but failed to position the paw correctly. It is important to note that at this age, the glycolytic muscles of *Sil1*^*wz*^ mice exhibit no macroscopic damage or alterations in the molecular markers linked to the progression of the pathology [18]. This defect likely reflects an early visible expression of the developing muscular damage.

Given the intellectual disability present in MSS patients [9], we explored the cognitive aspects of *Sil1*^*wz*^ mice using three different tests. The first test we employed for this evaluation was the nesting behaviour assessment. Similar to other tests that assess the preservation of everyday behaviours in rodents, such as the burrowing test, which incorporates tunnel maintenance and some aspects of defensive behaviours [55], the nesting building test provides insight into cognitive and behavioural function without the confounds of explicit training or reward contingencies [32, 56–58]. In the nest building test, *Sil1*^*wz*^ mice show a decrease in the total score compared to *Sil1*^*ht*^ mice at the 14^th^ week but not at the 10^th^ week of age. It is important to note that the overall score of the test is derived from the average of three parameters: paper movement within the cage relative to its original position, the extent of folding openings, and the gnawing percentage across the entire nesting material. We assume that evaluating these three parameters possibly excluded biases due to physical impairment in the *Sil1*^*wz*^ mice, as inferred by paper movement and fold opening. Conversely, the gnawing percentage is usually considered a measure of behavioural observation. *Sil1*^*wz*^ mice displayed a significant reduction in the average score compared to *Sil1*^*ht*^ mice, mainly given by the paper gnawing percentage. Finally, as we expected, female mice performed better than males for both the genotypes, consistent with their natural behaviour in nest building [59]. However, a limitation of this test could be represented by the choice of material used to build the nest, together with the environmental and strain-specific conditions, as well as a score dependent on the individual operator, which impacts the test results. The second test conducted, the NOR, showed no differences between the two experimental groups at either time points tested. Similarly, arm alternation in the Y-maze test revealed no differences between the two groups. The Y-maze test is commonly used to assess spatial working memory [60, 61], while the NOR test is designed to reveal deficits in learning and memory [62, 63]. These tests assess cognitive impairments linked to hippocampal and medial prefrontal cortex dysfunction, while minimising the confounding effects of physical performance demands inherent in tasks such as the Morris Water Maze or Barnes Maze [64–66].

The absence of deficits in these hippocampus-dependent tasks warrants consideration of several possibilities. First, this may indicate genuine sparing of hippocampal function in *Sil1*^*wz*^ mice at the ages examined, consistent with the predominantly cerebellar and muscular pathology that characterises MSS [9]. This would align with the clinical presentation of MSS, where motor dysfunction and cerebellar ataxia are the primary features, while cognitive impairment, though present, is more variable and may develop later or manifest differently than hippocampal-specific memory deficit*s* [9, 67, 68]. Second, compensatory mechanisms in hippocampal circuits may delay the onset of detectable cognitive dysfunction during the time window examined. The cerebellum degenerates as early as 6 weeks of age in this model [16, 19], while hippocampal and cortical regions may remain relatively preserved during early disease stages. Third, it is possible that the NOR and Y-maze tasks lack sufficient sensitivity to detect subtle hippocampal impairments that might emerge at later disease stages or require more challenging cognitive paradigms [60, 69]. The nesting behaviour test, which did reveal deficits at 14 weeks, may capture a broader spectrum of cognitive and neuropsychiatric dysfunction that is not solely hippocampus-dependent [58, 70]. Future studies employing more comprehensive cognitive batteries, or examining older animals, may clarify whether hippocampal dysfunction eventually manifests in this model.

Female *Sil1*^*wz*^ mice did not show differences in estrous cycle phase regularity with respect to female *Sil1*^*ht*^ mice. Estrous cycle phase did not significantly influence performance in motor or cognitive tests, mostly because these tasks primarily assess motor coordination and cognitive function rather than anxiety- or depression-related behaviours [26, 71, 72].

The correlation analysis of individual parameters from the tests assessing motor and cognitive functions in mice highlighted the robustness of the analysis and offered a clear distinction between the two experimental groups. The positive correlation between performance in the inverted screen and beam walking tests in *Sil1*^*wz*^ mice underlines that the onset of muscle damage follows the appearance of overt signs of cerebellar ataxia. The correlation between immobility on the inverted screen and beam walking performance likely reflects a shared underlying pathophysiology. Both tests assess motor coordination and muscle strength, which are progressively impaired in *Sil1*^*wz*^ mice due to cerebellar degeneration and myofibre abnormalities (Supplementary Fig. 10 and Supplementary Fig. 11). Specifically, performance on the inverted screen requires sustained grip strength and postural control, while beam walking depends on fine motor coordination and balance, both of which rely on the integrity of cerebellar function, which already begins to degenerate around the 6^th^ week of life, and neuromuscular integrity, which becomes impaired around the 10^th^ week of life. The positive correlation observed in *Sil1*^*wz*^ mice between impairments in these tasks suggests that mice with more severe cerebellar and muscular pathology exhibit deficits across multiple motor domains simultaneously, rather than isolated impairments in single functions. This pattern is consistent with the progressive nature of the disease, where animals with more advanced pathology show broader motor dysfunction. Regarding the *Sil1*^*ht*^ mice, an inverse correlation was observed between the number of times the animals detached their hindlimbs from the grid during the inverted screen test and the number of "missteps" in the beam walking test. This association reflects the absence of cerebellar ataxia and muscle fatigue in animals that do not exhibit the pathological phenotype.

Studying muscle function involves examining muscle histology, fibre diameter, the presence of adipocytes, and the extent of fibrotic tissue, all of which are correlated with muscle strength [73]. In our mice, we observed a clinical phenotype that mirrors what is observed in MSS patients [9, 26, 67, 68, 74–81], with woozy mice showing decreased muscle fibre size. Moreover, we observed the centralisation of nuclei within the fibres of the glycolytic muscles in *Sil1*^*wz*^ mice as early as the 30^th^ week of age. This difference was evident only in muscles with a glycolytic metabolic phenotype, such as the gastrocnemius and quadriceps, while no differences were observed in oxidative muscles, such as the soleus. We previously reported that *Sil1*^*wz*^ mice exhibit an irregular pattern of collagen fibres in the boundary between Achilles tendons and the soleus muscle with respect to *Sil1*^*ht*^ mice [82]. Our findings here align with prior reports in the *Sil1*^*Gt*^ model [23], which showed no differences in the soleus compared with wild-type animals at 3 months of age. One possible explanation for this phenomenon is related to the distinct metabolic profiles of these muscles. As previously reported [23], SIL1 loss affects the insulin receptor/IGF1 receptor signalling pathway and glucose metabolism, making glycolytic muscles like the gastrocnemius and quadriceps, which primarily rely on glucose, more susceptible to changes than the soleus.

We did not analyse muscle fibres separately by sex. The reason was twofold: preliminary analysis did not show significant differences, and fibre diameters are unlikely to exhibit sex-based differences in these muscles, since their phenotype is primarily influenced by age [83].

The molecular analysis performed on the quadriceps muscle confirmed increased levels of proteins associated with the unfolded protein response, such as BiP and pEIF2α, as well as those involved in the proteolysis process, such as Rab11 and LC3 [18]. In our previous study of patient-derived fibroblasts, we already observed the modulation of part of these markers [84]. Interestingly, here we found that while these markers were upregulated in the quadriceps, they showed similar levels between the two groups in the soleus. Molecular changes in the quadriceps provide key insights into the mechanisms driving disease progression. The upregulation of UPR markers (BiP, and pEIF2α) suggests persistent ER stress, which may compromise protein folding and promote apoptosis in glycolytic muscle [85, 86]. UPR activation may contribute to cellular damage because, in addition to the accumulation of misfolded proteins, it triggers the shutdown of protein translation by reducing levels of important proteins that are already in short supply due to protein misfolding. The concurrent elevation of Rab11 and the lipidated form of LC3 suggests dysregulation, or at least involvement, of both endosomal recycling and autophagy pathways. Rab11, a key regulator of endosomal trafficking and membrane receptor recycling, may be upregulated as a compensatory response to impaired protein trafficking and disrupted membrane homeostasi*s* [86, 87]. LC3 elevation indicates increased autophagosome formation, potentially reflecting an attempt to clear accumulated protein aggregates through autophagy [88]. However, if autophagic flux is impaired, as suggested by the progressive myofibre degeneration, this could contribute to toxic protein accumulation rather than clearance [89, 90].

In conclusion, using motor tests, histological analysis, and a molecular approach, we observed in these mice similar muscular deficits present in MSS patients. In addition to the overt cerebellar damage identified through coordination and balance tests (accelerating rotarod, beam walking, and pole test), muscular atrophy was highlighted by the inverted screen test. The animals exhibited longer durations of immobility and a significantly reduced frequency of hindlimb detachment from the grid. The reduced muscle fibre size also reflects a characteristic commonly observed in human patients, such as the decrease in muscle mass due to the loss of muscle fibres. Finally, in the same muscle, there is a reduction in creatine kinase B levels, an enzyme crucial for energy transduction in tissues with high demand and also involved in brain energy metabolism [91]. In MSS patients, a moderately increased serum creatine kinase concentration was observed [9, 67, 68, 92]. However, it is important to note that we investigated the B-isoform of the enzyme, which is the brain-type isoenzyme, primarily reflecting brain activity rather than skeletal muscle function.

Beyond the proteostatic mechanisms examined here, epigenetic regulation may contribute to the differential vulnerability observed between muscle types. DNA methylation, histone modifications, and miRNA expression patterns could influence stress response gene expression and modulate disease progression in a tissue-specific manner [93]. Future investigations employing epigenetic profiling and transcriptomic analyses in both mouse models and patient samples could reveal novel regulatory mechanisms underlying the selective vulnerability of glycolytic muscles and identify potential therapeutic targets.

Compared to previous studies using this animal model, we incorporated a cognitive characterisation to investigate the brain functional disturbances commonly observed in MSS patients. For the first time, we incorporated sex and hormone evaluations in these tests to prevent sex-biased data interpretation. Furthermore, we explored the molecular mechanisms involved in the soleus, a muscle with oxidative metabolism that had not previously been investigated in this model at the molecular level [23]. The observation that female *Sil1*^*wz*^ animals performed better than male *Sil1*^*wz*^ animals suggests that females may exhibit distinct patterns of variation, which future studies should explore more closely. Finally, investigating the pathways involved in maintaining structural health in oxidative muscles compared to glycolytic ones could provide a viable approach for discovering new therapeutic methods, particularly from a palliative perspective for affected patients.

## Study Limitations

The lack of multiple time points to assess the animals histologically and molecularly prevents us from precisely determining when perturbations occur in the different tissues. In line with previous studies, we used *Sil1*^*ht*^ mice as controls to assess the motor performance and cognitive behaviour of *Sil1*^*wz*^ mice [22]. For the endpoints examined in this study, the *Sil1*^*ht*^ mice provide an appropriate reference point to evaluate the severe phenotype of *Sil1*^*wz*^ mice, while the monoallelic expression in *Sil1*^*ht*^ mice was not associated with a pathological phenotype, consistent with mouse and human studies showing that heterozygous carriers of *SIL1* mutations do not display any pathological phenotype [9, 94]. Wild-type controls would be particularly important for future studies involving deeper molecular investigations, such as gene expression regulatory mechanisms that could be influenced by the monoallelic expression of the *Sil1* gene in *Sil1*^*ht*^ animals. Another potential limitation of the study lies in the molecular analyses, as they were performed on a muscle different from the one used to establish macroscopic differences through the histological approach. We chose to use the gastrocnemius and soleus muscles for histological analyses because they were collected and processed as a single sample, avoiding potential variability introduced by isolation and sample preparation procedures. It is also important to specify that both the gastrocnemius and quadriceps are glycolytic muscles. Therefore, for the molecular analyses, we used quadriceps to align with findings from previous studies.

## Supplementary Information

Below is the link to the electronic supplementary material.

Supplementary file1 (DOCX 8.00 MB)

## Data Availability

No datasets were generated or analysed during the current study.
