# Correction: Systematic Phenotyping and Molecular Analysis of the Woozy Mouse: A Preclinical Model of Cerebellar Ataxia

**DOI:** 10.1007/s12035-026-05695-1

**Published:** 2026-02-02

**Authors:** Fabio Bellia, Laura Amodei, Anna Giulia Ruggieri, Francesca Potenza, Marianna Viele, Manuela Bomba, Francesco Del Pizzo, Manuela Iezzi, Alberto Granzotto, Luca Federici, Michele Sallese

**Affiliations:** 1 Center for Advanced Studies and Technology (CAST), “G. d’Annunzio” University of Chieti-Pescara, 66100 Chieti, Italy; 2 Department of Innovative Technologies in Medicine and Dentistry, “G. d’Annunzio” University of Chieti-Pescara, 66100 Chieti, Italy; 3 Department of Neuroscience, Imaging, and Clinical Sciences, “G. d’Annunzio” University of Chieti-Pescara, 66100 Chieti, Italy

**Correction: Molecular Neurobiology (2025) 63:258**

10.1007/s12035-025-05577-y

In the originally published version of this article, Figure 8a was incorrectly labelled. As a consequence, the quantifications shown in Figure 8b and c, a paragraph in the Results section, and Supplementary Table 10 contained incorrect information derived from a nonspecific band.

The incorrect elements are reported below for transparency, followed by the corrected Figure 8, revised Results paragraph, and corrected Supplementary Table 10.

**Incorrect Figure 8** 
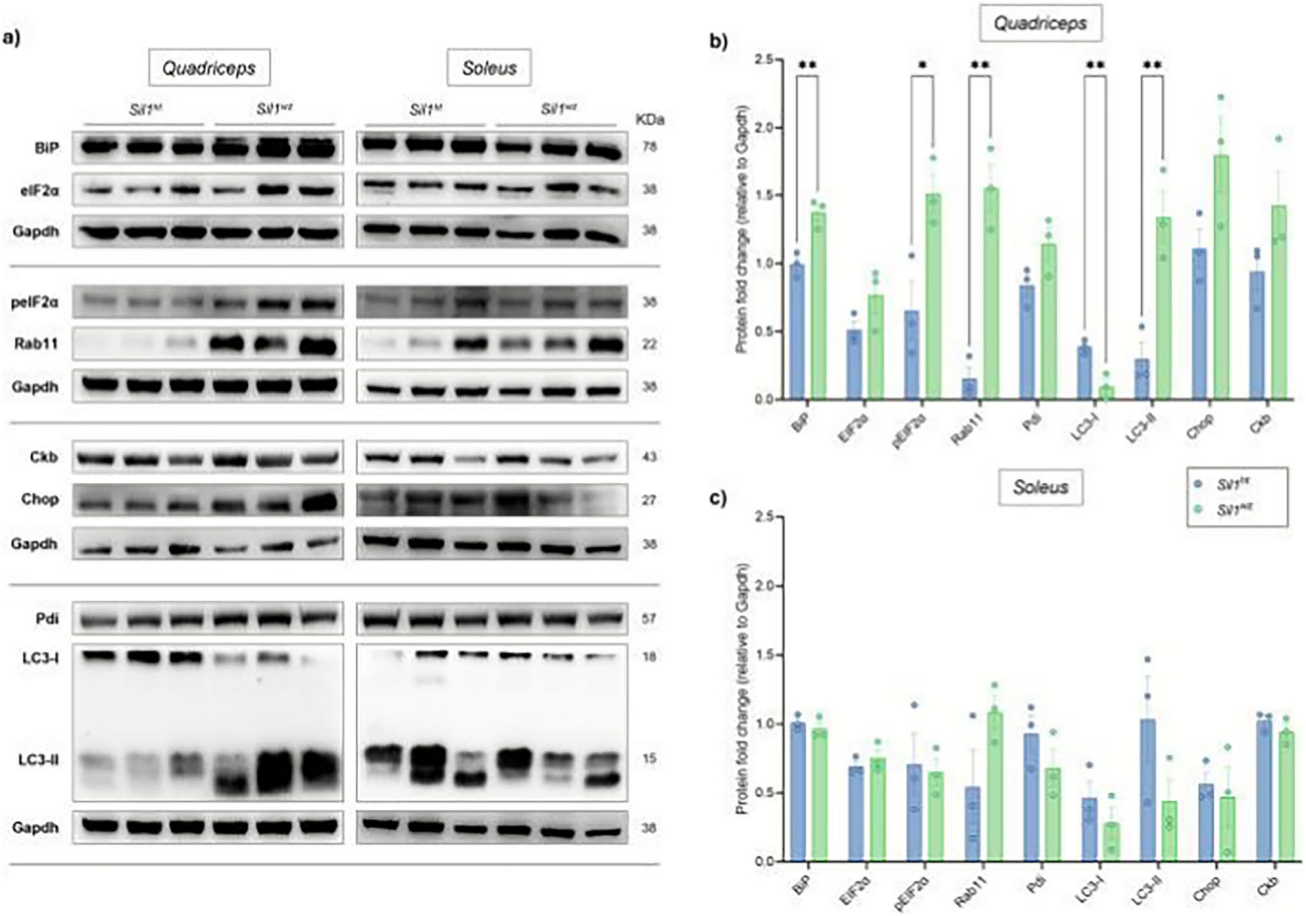


**Incorrect paragraph of the result**

Western blot analysis in the quadriceps of 26-week-old *Sil1*^*wz*^ mice revealed increased protein levels of BiP (*Sil1*^*wz*^: 1.37 ± 0.06, *Sil1*^*ht*^: 0.99 ± 0.05; *p* = 0.0093), pEIF2α (*Sil1*^*wz*^: 1.51 ± 0.14, *Sil1*^*ht*^: 0.65 ± 0.21; *p* = 0.0279), Rab11 (*Sil1*^*wz*^: 1.55 ± 0.17, *Sil1*^*ht*^: 0.16 ± 0.08; *p* = 0.0019), and the lipidated form of LC3 (*Sil1*^*wz*^: 1.34 ± 0.19, *Sil1*^*ht*^: 0.30 ± 0.12; *p* = 0.0095), along with decreased levels of the non-lipidated form of LC3 (*Sil1*^*wz*^: 0.10 ± 0.05, *Sil1*^*ht*^: 0.39 ± 0.03; *p* = 0.0093) compared to *Sil1*^*ht*^ mice (Fig. 8a and b, and Supplementary Table 10). In the soleus of the same mice, we observed a tendency toward increased Rab11 levels (*Sil1*^*wz*^: 1.09 ± 0.12, *Sil1*^*ht*^: 0.55 ± 0.27), together with decreased Pdi (*Sil1*^*wz*^: 0.68 ± 0.14, *Sil1*^*ht*^: 0.93 ± 0.13) and the lipidated form of LC3 (*Sil1*^*wz*^: 0.44 ± 0.16, *Sil1*^*ht*^: 1.03 ± 0.31) levels (Fig. 8a and c, and Supplementary Table 10). However, no statistical significance was reached between the two experimental groups in the soleus (see Supplementary Table 10).

**Incorrect Supplementary Table 10**

**Supplementary Table 10:**

**Table:** 

**Protein**	**Quadriceps** **Mean ± SEM**			**Soleus** **Mean ± SEM**		
	***Sil1***^***ht***^	***Sil1***^***wz***^	*P* value	***Sil1***^***ht***^	***Sil1***^***wz***^	*P* value
**BiP**	0.99 ± 0.05	1.37 ± 0.06	**0.0093**	1.01 ± 0.03	0.97 ± 0.05	0.4558
**EIF2α**	0.51 ± 0.06	0.78 ± 0.13	0.1605	0.69 ± 0.04	0.75 ± 0.06	0.4545
**pEIF2α**	0.65 ± 0.21	1.51 ± 0.14	**0.0279**	0.71 ± 0.23	0.65 ± 0.10	0.8204
**Rab11**	0.16 ± 0.08	1.55 ± 0.17	**0.0019**	0.55 ± 0.27	1.09 ± 0.12	0.1384
**Pdi**	0.84 ± 0.08	1.14 ± 0.12	0.1090	0.93 ± 0.13	0.68 ± 0.14	0.2598
**LC3-I**	0.39 ± 0.03	0.10 ± 0.05	**0.0093**	0.46 ± 0.12	0.28 ± 0.11	0.3377
**LC3-II**	0.30 ± 0.12	1.34 ± 0.19	**0.0095**	1.03 ± 0.31	0.44 ± 0.16	0.1651
**Chop**	1.11 ± 0.14	1.80 ± 0.28	0.0939	0.57 ± 0.09	0.47 ± 0.22	0.7072
**Ckb**	0.94 ± 0.14	1.47 ± 0.25	0.1581	1.02 ± 0.04	0.95 ± 0.06	0.3270

**Corrected Figure 8** 
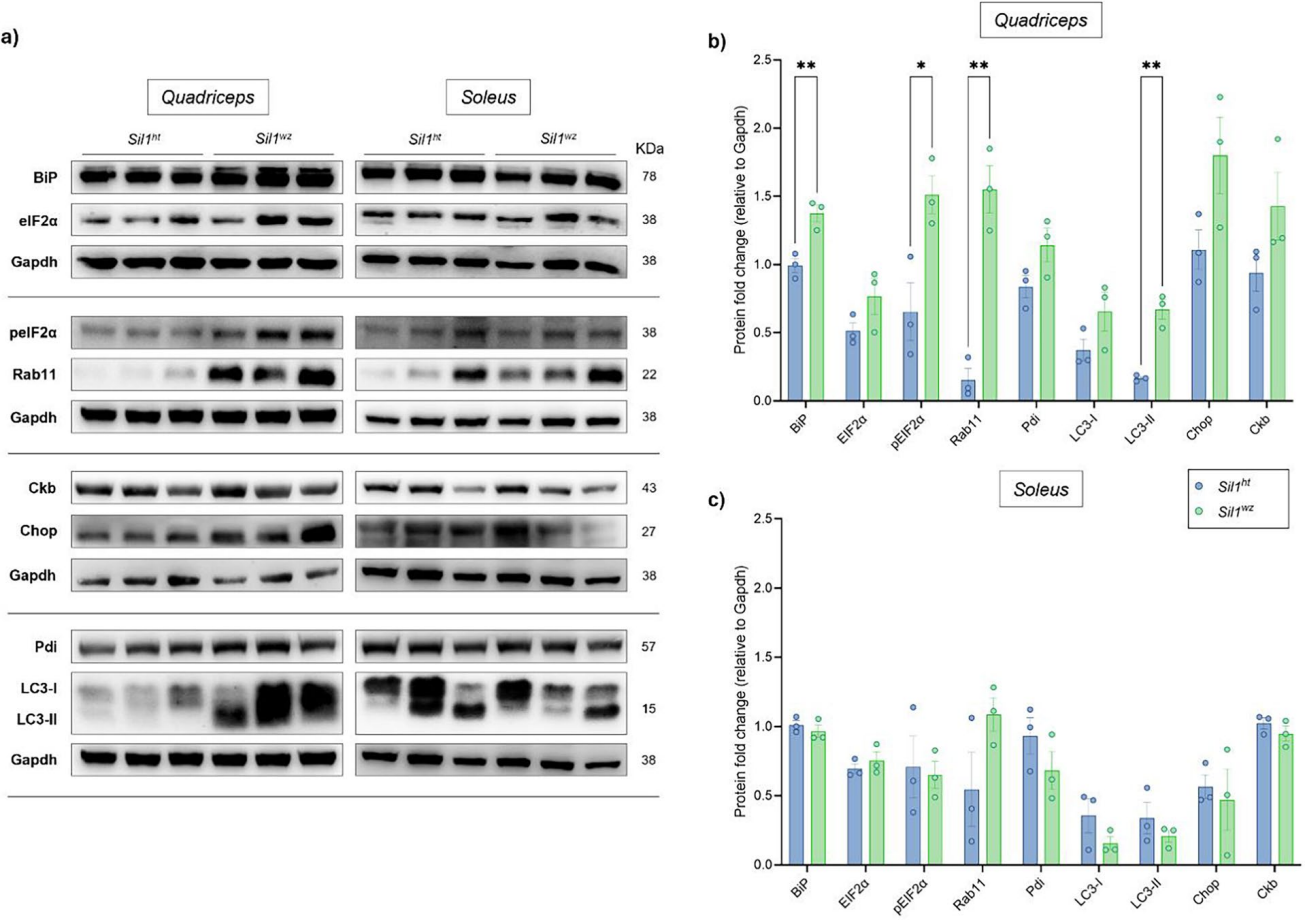


**Correct paragraph of the result**

Western blot analysis in the quadriceps of 26-week-old *Sil1*^*wz*^ mice revealed increased protein levels of BiP (*Sil1*^*wz*^: 1.37 ± 0.06, *Sil1*^*ht*^: 0.99 ± 0.05; *p* = 0.0093), pEIF2α (*Sil1*^*wz*^: 1.51 ± 0.14, *Sil1*^*ht*^: 0.65 ± 0.21; *p* = 0.0279), Rab11 (*Sil1*^*wz*^: 1.55 ± 0.17, *Sil1*^*ht*^: 0.16 ± 0.08; *p* = 0.0019), and the lipidated (LC3-II) form of LC3 compared to *Sil1*^*ht*^ mice (LC3-II: *Sil1*^*wz*^: 0.67 ± 0.07, *Sil1*^*ht*^: 0.17 ± 0.01; *p* = 0.0022) (Fig. 8a and b, and Supplementary Table 10). In the soleus of the same mice, we observed a tendency toward increased Rab11 levels (*Sil1*^*wz*^: 1.09 ± 0.12, *Sil1*^*ht*^: 0.55 ± 0.27), together with decreased Pdi (*Sil1*^*wz*^: 0.68 ± 0.14, *Sil1*^*ht*^: 0.93 ± 0.13) and both forms of LC3 (LC3-I: *Sil1*^*wz*^: 0.16 ± 0.05, *Sil1*^*ht*^: 0.36 ± 0.12; LC3-II: *Sil1*^*wz*^: 0.21 ± 0.04, *Sil1*^*ht*^: 0.34 ± 0.11) levels (Fig. 8a and c, and Supplementary Table 10). However, no statistical significance was reached between the two experimental groups in the soleus (see Supplementary Table 10).

**Corrected Supplementary Table 10**

**Supplementary Table 10:**

**Table:** 

**Protein**	**Quadriceps** **Mean ± SEM**			**Soleus** **Mean ± SEM**		
	***Sil1***^***ht***^	***Sil1***^***wz***^	*P* value	***Sil1***^***ht***^	***Sil1***^***wz***^	*P* value
**BiP**	0.99 ± 0.05	1.37 ± 0.06	**0.0093**	1.01 ± 0.03	0.97 ± 0.05	0.4558
**EIF2α**	0.51 ± 0.06	0.78 ± 0.13	0.1605	0.69 ± 0.04	0.75 ± 0.06	0.4545
**pEIF2α**	0.65 ± 0.21	1.51 ± 0.14	**0.0279**	0.71 ± 0.23	0.65 ± 0.10	0.8204
**Rab11**	0.16 ± 0.08	1.55 ± 0.17	**0.0019**	0.55 ± 0.27	1.09 ± 0.12	0.1384
**Pdi**	0.84 ± 0.08	1.14 ± 0.12	0.1090	0.93 ± 0.13	0.68 ± 0.14	0.2598
**LC3-I**	0.37 ± 0.08	0.65 ± 0.14	0.1601	0.36 ± 0.12	0.16 ± 0.05	0.2060
**LC3-II**	0.17 ± 0.01	0.67 ± 0.07	**0.0022**	0.34 ± 0.11	0.21 ± 0.04	0.3545
**Chop**	1.11 ± 0.14	1.80 ± 0.28	0.0939	0.57 ± 0.09	0.47 ± 0.22	0.7072
**Ckb**	0.94 ± 0.14	1.47 ± 0.25	0.1581	1.02 ± 0.04	0.95 ± 0.06	0.3270

These corrections do not affect the interpretation of the results or the conclusions of the study. The authors apologize for the error and any inconvenience it may have caused.

The original article has been corrected.

