# Supplementary material for: Systematic Phenotyping and Molecular Analysis of the Woozy Mouse: A Preclinical Model of Cerebellar Ataxia
Source: Mol Neurobiol. 2025 Dec 6;63(1):258. doi: 10.1007/s12035-025-05577-y (PMC12680694; doi:10.1007/s12035-025-05577-y)
Supplement: Supplementary file 1 — Supplementary file1 (DOCX 8.00 MB) [file 12035_2025_5577_MOESM1_ESM.docx]

Supplementary materials

**Systematic Phenotyping and Molecular Analysis of the Woozy Mouse: A Preclinical Model of Cerebellar Ataxia**

Bellia Fabio**^1,2^**, Amodei Laura**^1,2^**, Ruggieri Anna Giulia**^1,2^**, Potenza Francesca**^1,2^**, Viele Marianna**^1,2^**, Bomba Manuela**^1,3^**, Del Pizzo Francesco**^1,3^**, Iezzi Manuela**^1,3^**, Granzotto Alberto**^1,3^**, Federici Luca**^1,2^**, Sallese Michele**^1,2^**.

**^1^** Center for Advanced Studies and Technology (CAST), “G. d’Annunzio” University of Chieti-Pescara, 66100 Chieti, Italy.

**^2^** Department of Innovative Technologies in Medicine and Dentistry, “G. d’Annunzio” University of Chieti-Pescara, 66100 Chieti, Italy.

**^3^** Department of Neuroscience, Imaging, and Clinical Sciences, “G. d'Annunzio” University, Chieti-Pescara, Italy.

*Corresponding author: Michele Sallese Ph.D

E-mail: [michele.sallese@unich.it](mailto:michele.sallese@unich.it)

**Supplementary Figure 1**: (**a**) Schematic pictures of the main observations made to determine the individual estrous cycle’s phase. Proestrus: mostly nucleated cells, with few cornified cells; Estrous: big abundance of cornified cells as the main clearly visible cell population during this phase; Metestrus: characterized by the presence of nucleated, cornified cells, but also a big abundance of leukocytes; Diestrus: leukocytes are the most representative cell population in this phase but nucleated and cornified cells are also visible. Scale bar corresponds to 250 µm). (**b**) Graphical representation of the distribution of individual animals by estrus stage over time and across tests.

**Supplementary Figure 2**: Latency to fall in *Sil1^ht^* (**a**) and *Sil1^wz^* (**b**) mice. Data are represented as scattered dot plots (mean ± SEM, of each group) and expressed as the percentage of the maximum time for the individual animal. Males = blue circles; Females = red circles. Significant differences are indicated (*p<0.05).

**Supplementary Figure 3**: Time to traverse the bar and number of contralateral falls in *Sil1^ht^* (**a**, **b**) and *Sil1^wz^* (**c**, **d**) mice. Data are represented as scattered dot plots (mean ± SEM, of each group). Males = blue circles; Females = red circles.

**Supplementary Figure 4**: Latency to fall, number of episodes, and immobility time for the individual parameter scored in *Sil1^ht^* (**a**, **c**), and *Sil1^wz^* (**b**, **d**) mice. Data are represented as scattered dot plots (mean ± SEM, of each group). Males = blue circles; Females = red circles.

**Supplementary Figure 5**: Paper movement, degree of opening, and gnawing percentage observed in the nesting building at the 10^th^ and 14^th^ week of life in the overall (**a**), male (**b**), and female (**c**), *Sil1^ht^* (**d**), and *Sil1^wz^* mice (**e**). Data are represented as scattered dot plots (mean ± SEM, of each group). *Sil1^wz^* = green circles; *Sil1^ht^* = blue circles; Males = blue circles; Females = red circles.

**Supplementary Figure 6**: Discrimination index observed in the Novel object recognition test at the 5^th^ and 14^th^ week of life in the overall (**a**), male (**b**), and female (**c**), *Sil1^ht^* (**d**), and *Sil1^wz^* mice (**e**). Data are represented as scattered dot plots (mean ± SEM, of each group). *Sil1^wz^* = green circles; *Sil1^ht^* = blue circles; Males = blue circles; Females = red circles.

**Supplementary Figure 7**: Percentage of alternation observed in Y-maze test at the 10^th^ and 14^th^ week of life in the overall (**a**), male (**b**), and female (**c**), *Sil1^ht^* (**d**), and *Sil1^wz^* mice (**e**). Data are represented as scattered dot plots (mean ± SEM, of each group). *Sil1^wz^* = green circles; *Sil1^ht^* = blue circles; Males = blue circles; Females = red circles.

**Supplementary Figure 8**: Cross-sectional area (CSA - µm^2^) of gastrocnemius (**a**) and soleus (**b**) muscular fibres measured in 26-week-old *Sil1^ht^* and *Sil1^wz^* mice. Data are represented as scattered dot plots (mean ± SEM, of each group). Male *Sil1^wz^* = green circles; male *Sil1^ht^* = blue circles; female *Sil1^wz^* = red circles; female *Sil1^ht^* = orange circles. Significant differences are indicated (****p<0.001).

**Supplementary Figure 9**: Body weight across the first 25 weeks of life in male and female *Sil1^ht^* and *Sil1^wz^* mice (**a**). Weekly weight gain (% respect the previous week) in male (**b**) and female (**c**) *Sil1^ht^* and *Sil1^wz^* mice.

**Supplementary Figure 10**: Hematoxylin & Eosin staining of quadriceps cross sections of 16-week-old (**a**), 22-week-old (**b**), and 33-week-old (**c**) *Sil1^wz^* mice. White-filled triangles indicate nuclear accumulation between the muscular fibres; black-filled triangles indicate myonuclei centralization.

**Supplementary Figure 11**: Hematoxylin & Eosin staining of longitudinal cerebellum slices in 26-week-old *Sil1^ht^* (**a**, **b**, and **c**) and *Sil1^wz^* (**d**, **e**, and **f**) mice. Black-filled triangles indicate Purkinje cells (PCs). Differences are visible between the neocerebellum (lobules I-VIII – **b** and **e**) and vestibulocerebellum (lobules IX-X – **c** and **f**).

**Supplementary Table 1 –** List of the Abs used for the Western blot analysis.

**Supplementary Table 2 –** Accelerating rotarod test results. Group means ± SEM of the individual week are reported. P values statistically significant are highlighted in bold (Mann-Whitney test with Holm-Sidak post hoc correction).

**Supplementary Table 3 –** Beam walking test results. Group means ± SEM of the individual week are reported for both the time to traverse the beam and the number of missteps. P values statistically significant are highlighted in bold (Mann-Whitney test with Holm-Sidak post hoc correction).

**Supplementary Table 4 –** Pole test results. Group means ± SEM of the t-turn and t-total displayed at the 14^th^ and 16^th^ week are reported. P values statistically significant are highlighted in bold (Mann-Whitney test with Holm-Sidak post hoc correction).

**Supplementary Table 5 –** Inverted screen test results. Group means ± SEM of the latency time to fall, immobility time (s), immobility (n), forelimbs, and hindlimbs at the 16^th^ week are reported. P values statistically significant are highlighted in bold (Mann-Whitney test with Holm-Sidak post hoc correction).

**Supplementary Table 6 –** Nesting building test results. Group means ± SEM of the individual score assigned for the paper movement, the opening, and gnawing are reported. P values statistically significant are highlighted in bold (Mann-Whitney test with Holm-Sidak post hoc correction).

**Supplementary Table 7 –** Novel object recognition test results observed during the 5^th^ and 14^th^ weeks. Data are reported as group mean (s) ± SEM.

**Supplementary Table 8 –** Y-maze test results observed during the 5^th^ and 14^th^ weeks. Data are reported as group mean (s) ± SEM.

**Supplementary Table 9 –** Spearman’s correlation analysis between the individual values of motor (Rotarod, Beam walking, Inverted screen, Pole) and cognitive (Nesting) tests considering the overall population, *Sil1^ht^*, and *Sil1^wz^* mice. Cells in the upper part represent Spearman’s r; cells in the lower part represent p values. BW = Beam Walking; IS = Inverted screen test; PT = Pole test; NB = Nesting building test.

**Supplementary Table 10 –** Proteins level quantification in the quadriceps and soleus of 26-week-old *Sil1^ht^*, and *Sil1^wz^* mice. Data are reported as group mean (s) ± SEM. P values are indicated. Statistically significant results (Unpaired t-test) are highlighted in bold.

**Supplementary Table 11** – Cross sectional area (µm^2^) in the gastrocnemius and soleus of 26-week-old *Sil1^ht^* and *Sil1^wz^* mice, stratified by sex. Statistically significant results (Mann-Whitney t-test) are highlighted in bold.

**Supplementary Figure 1**:


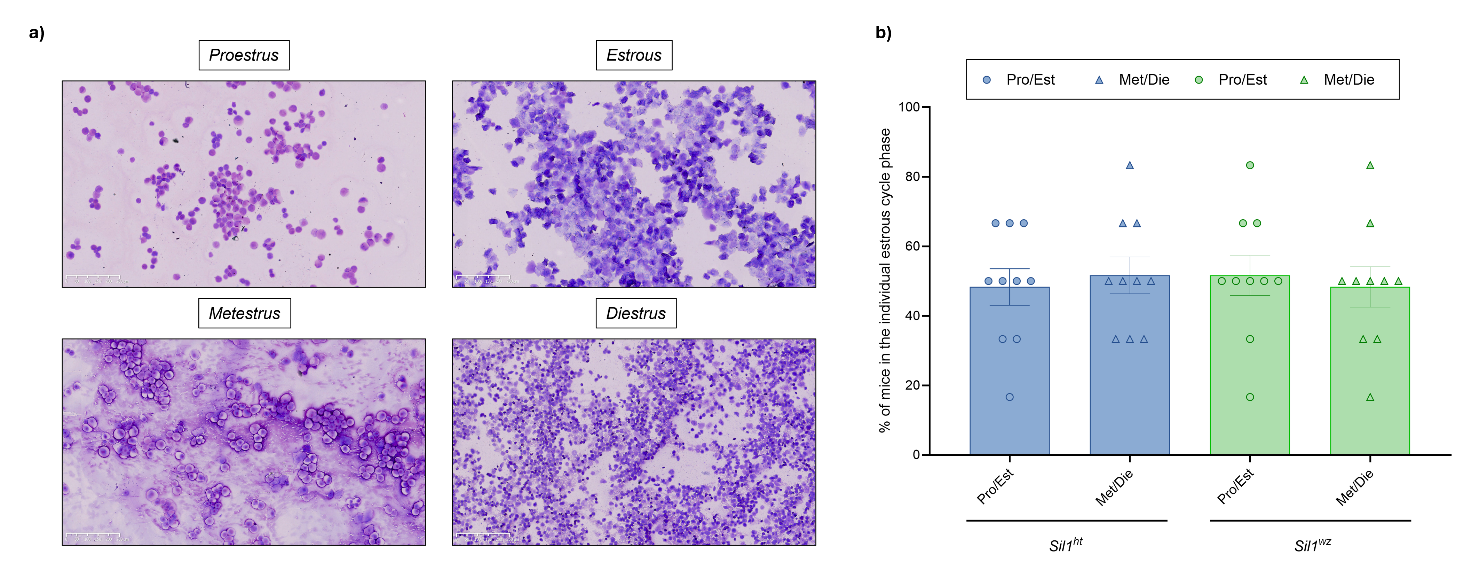


**Supplementary Figure 2**:


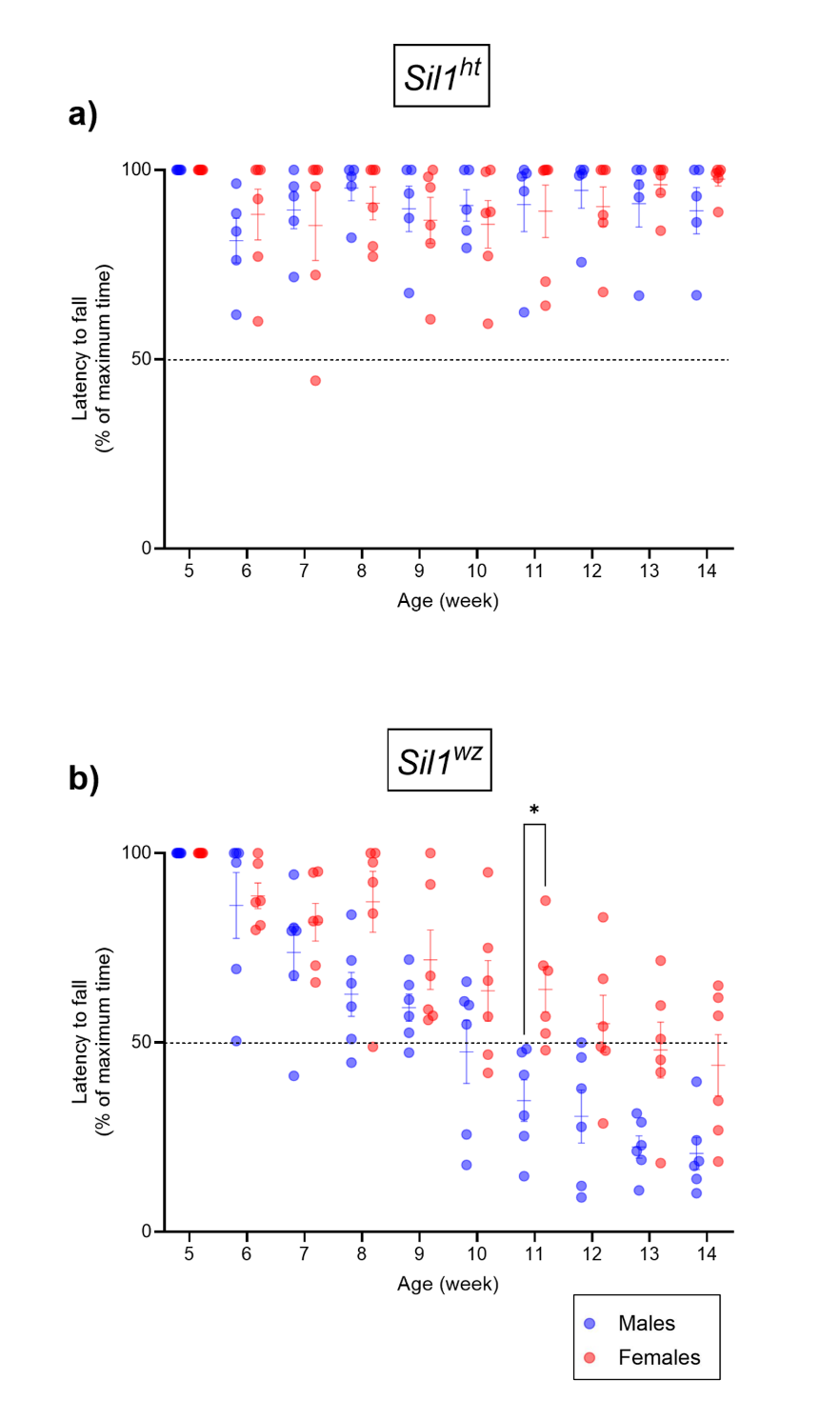


**Supplementary Figure 3**:


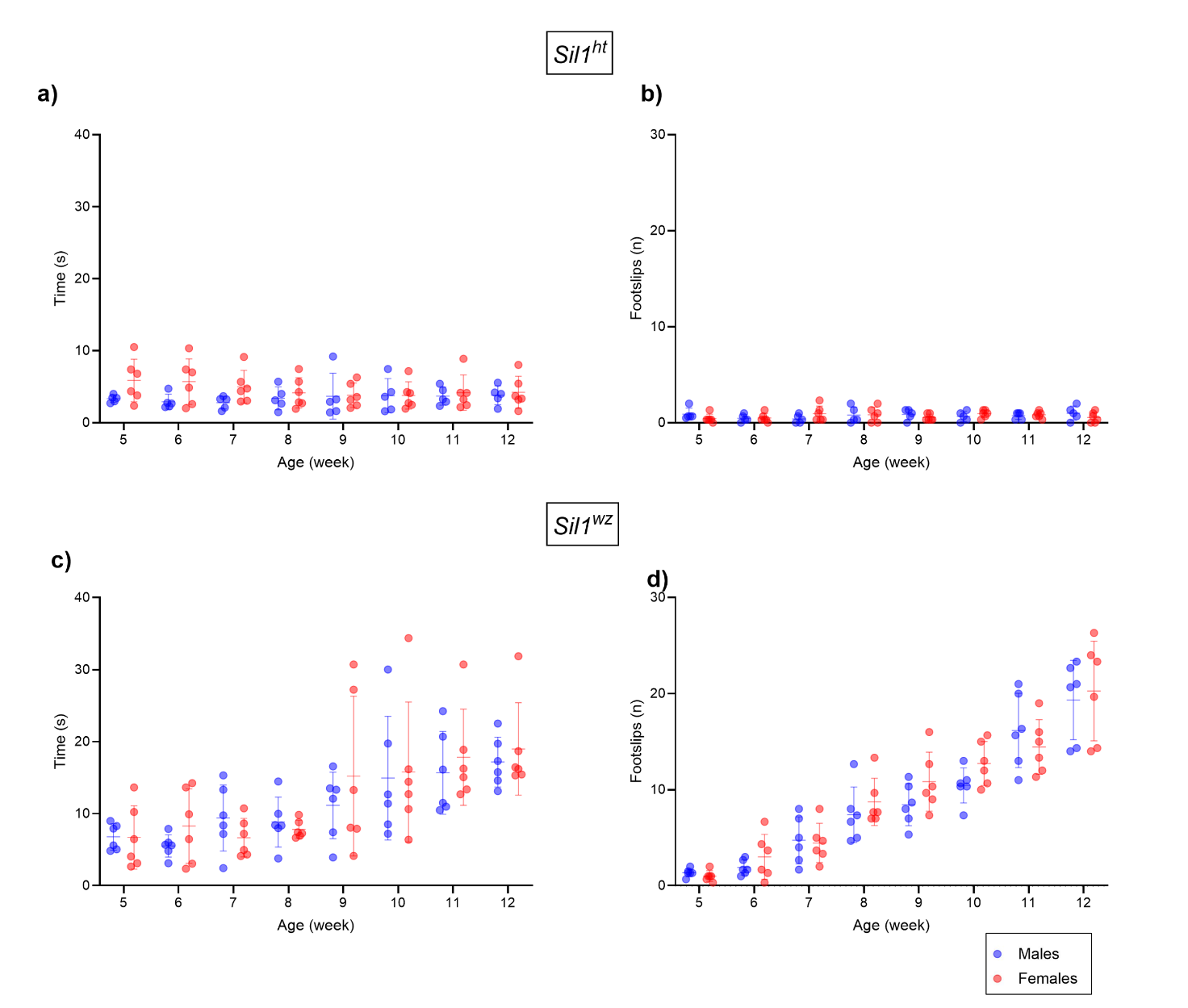


**Supplementary Figure 4**:


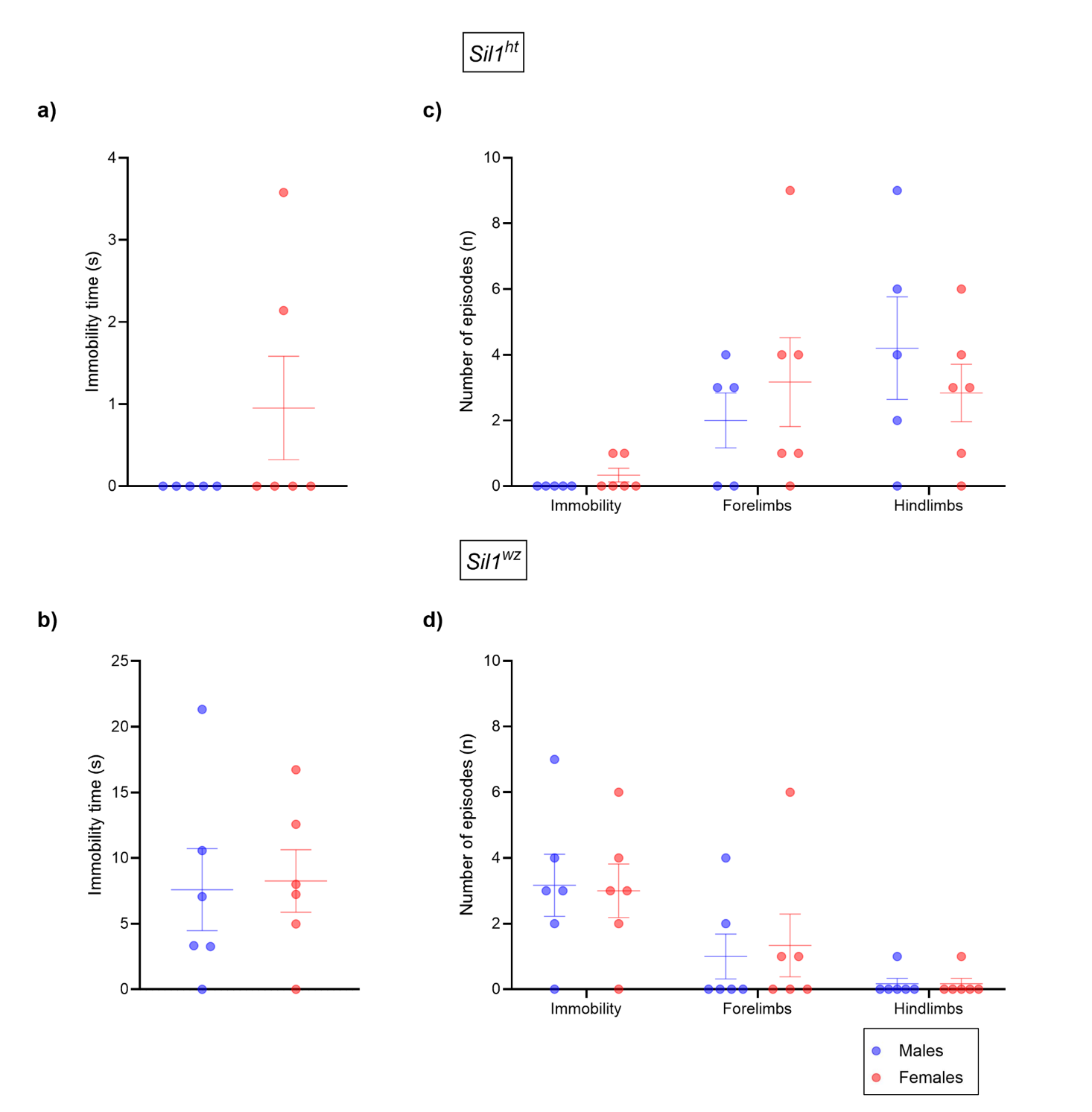


**Supplementary Figure 5**:


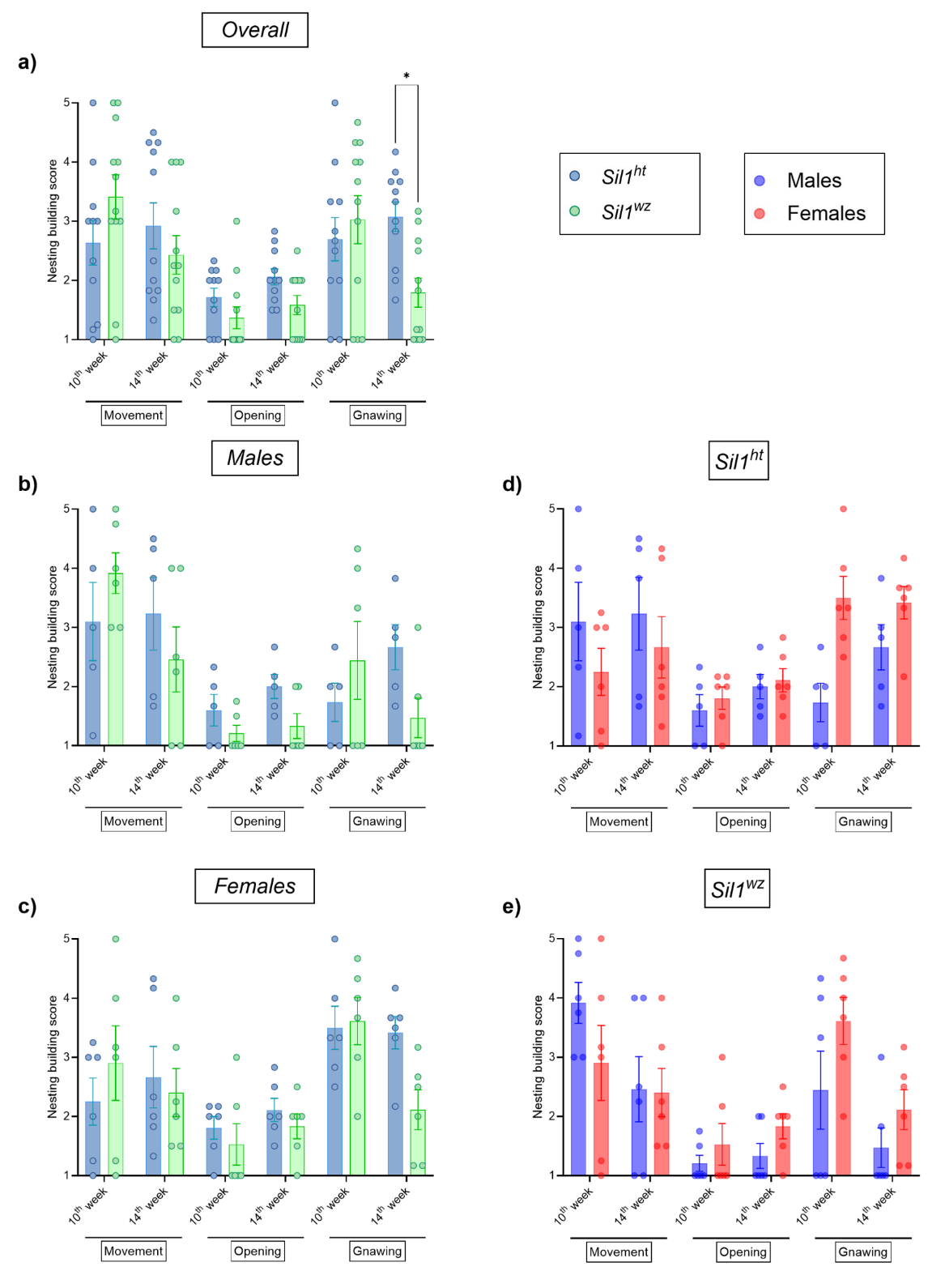


**Supplementary Figure 6**:


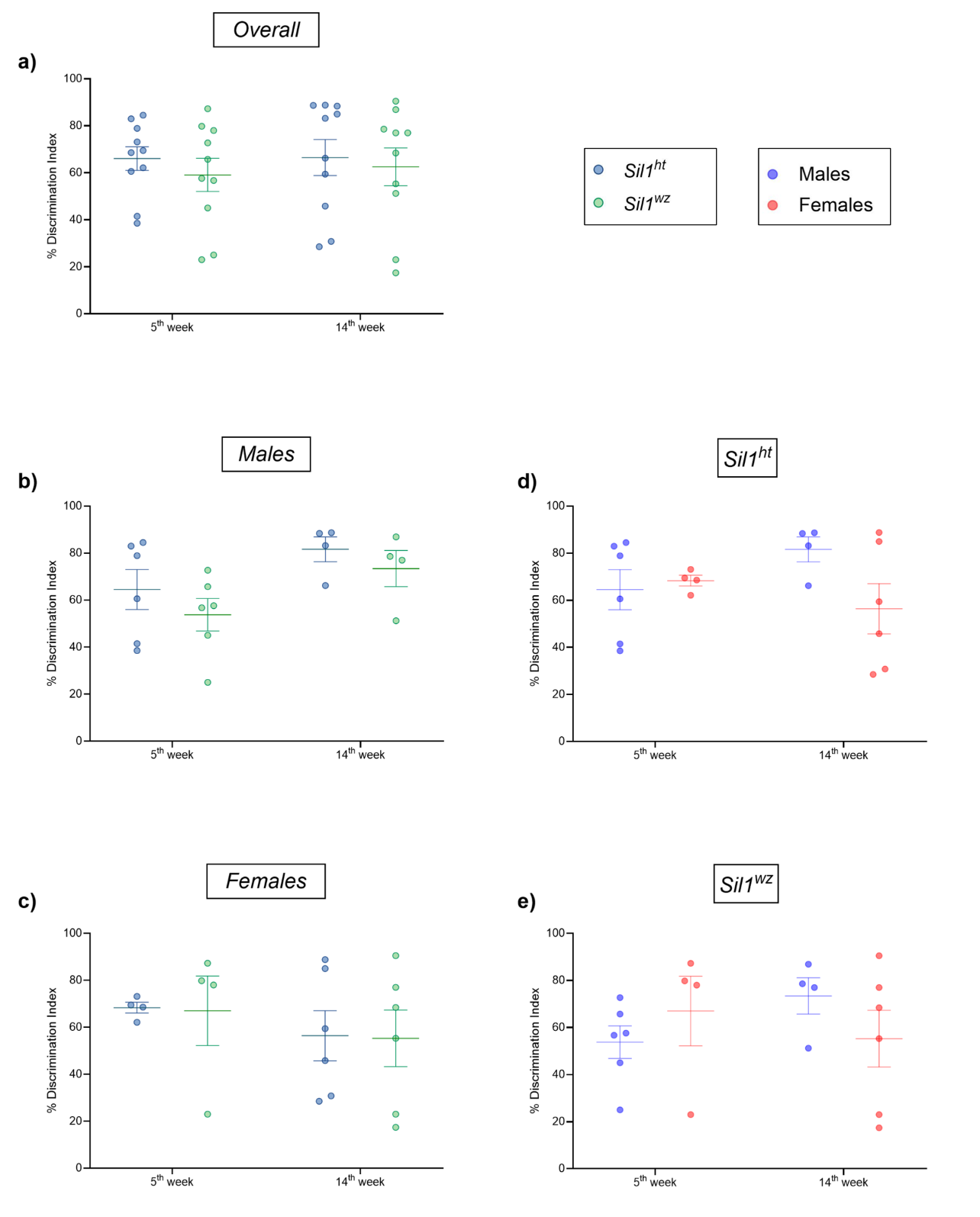


**Supplementary Figure 7**:


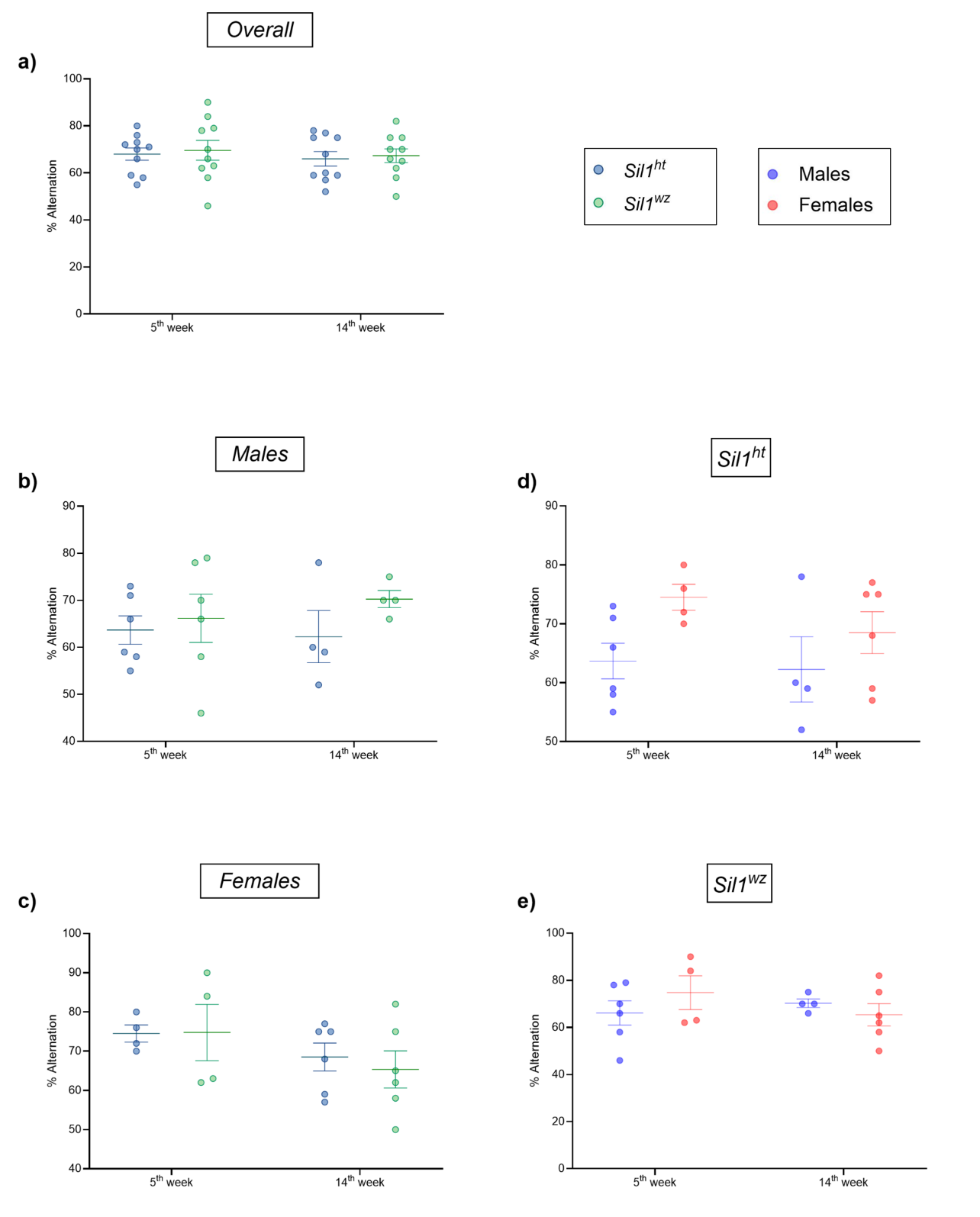


**Supplementary Figure 8**:


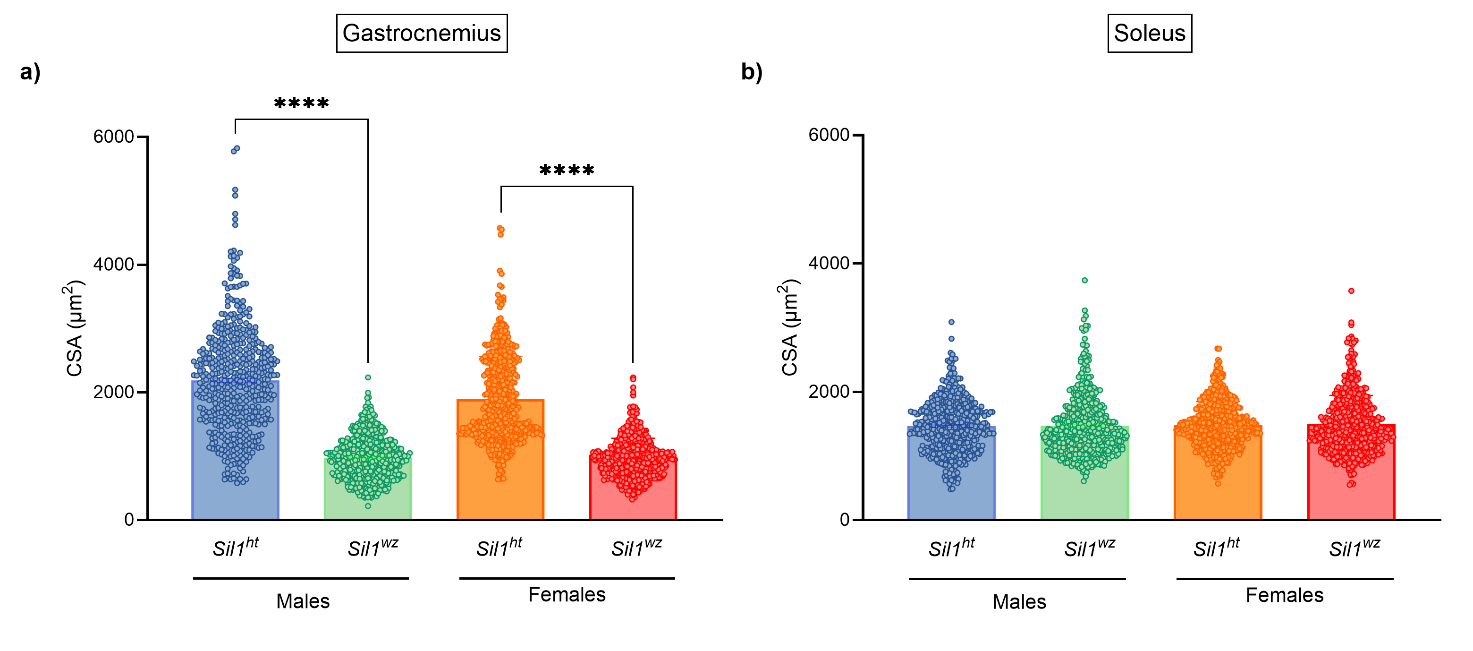


**Supplementary Figure 9**:


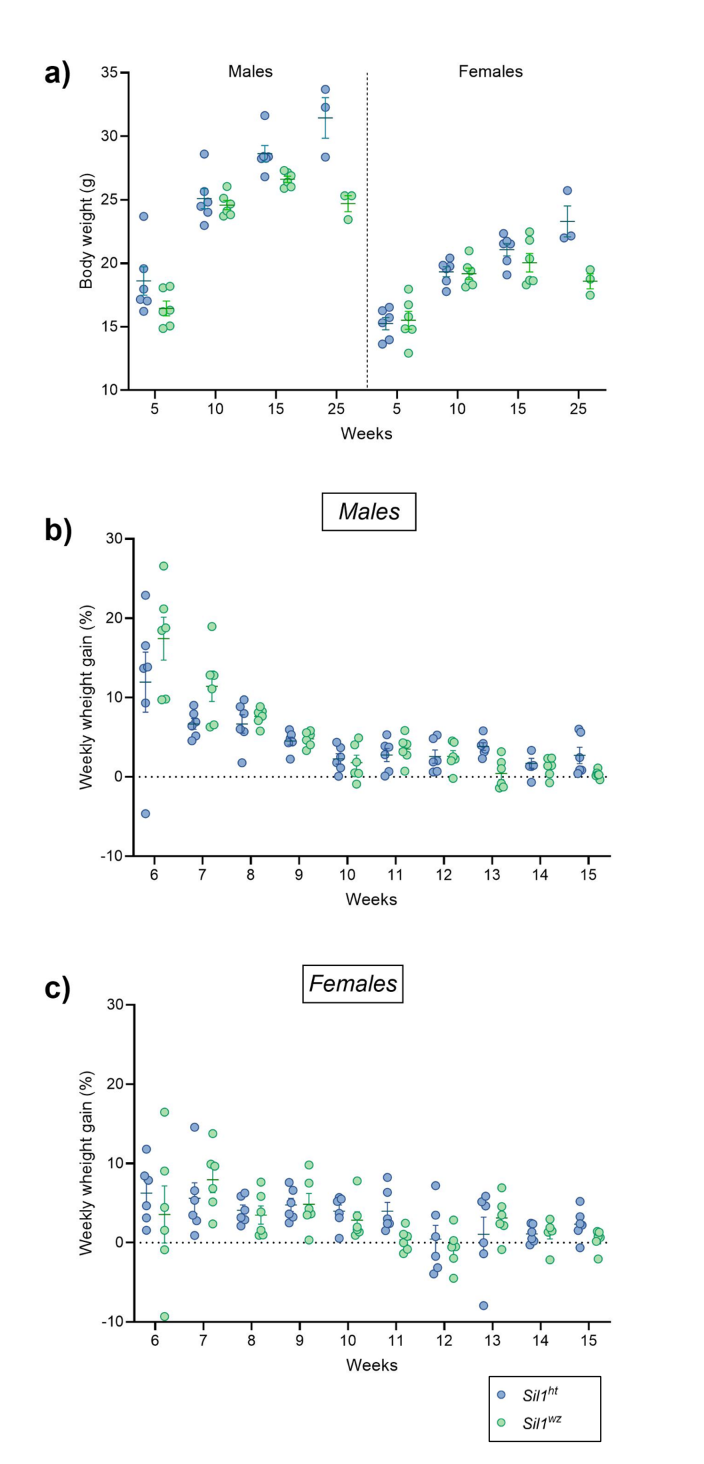


**Supplementary Figure 10**:


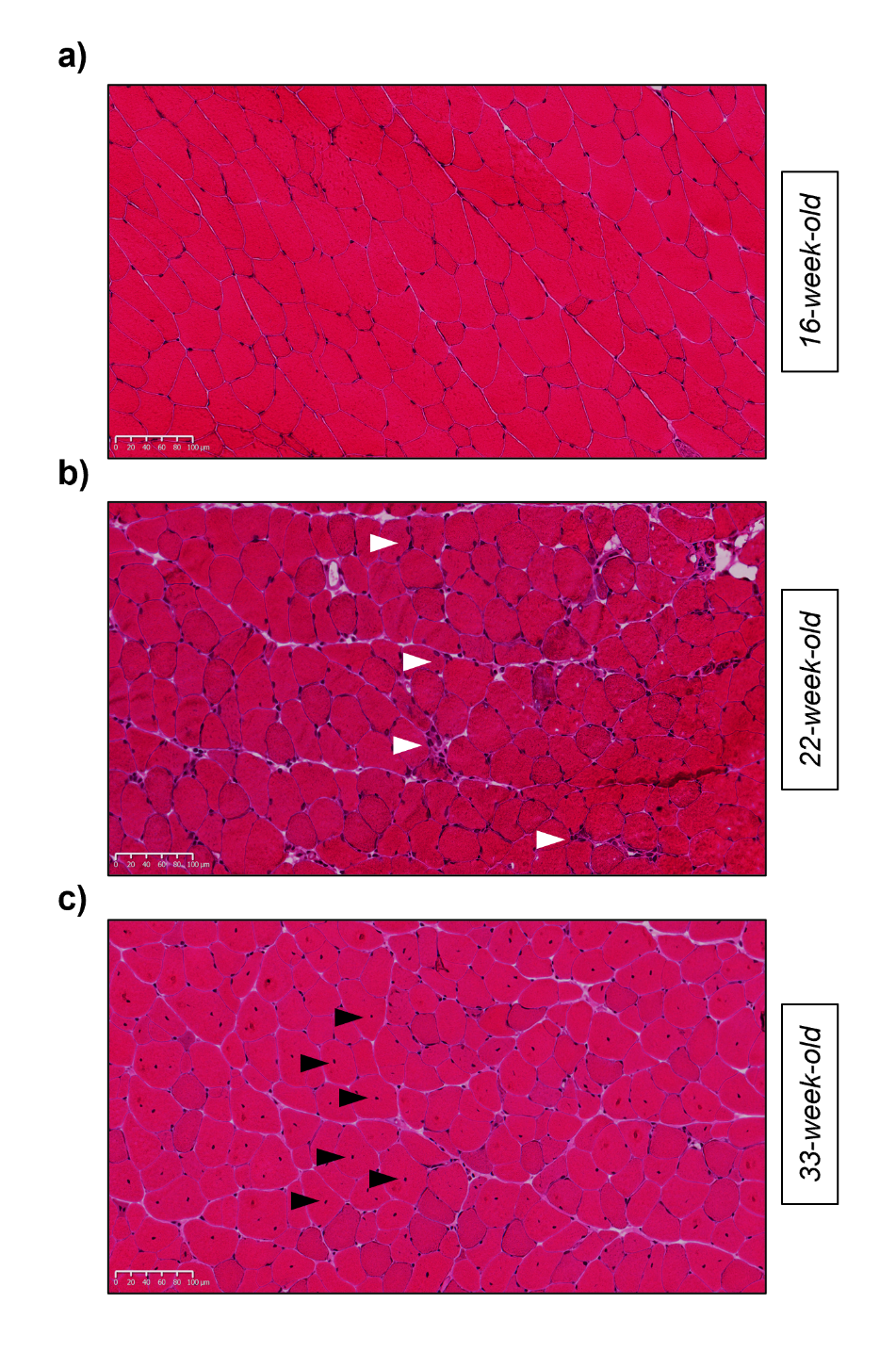


**Supplementary Figure 11**:


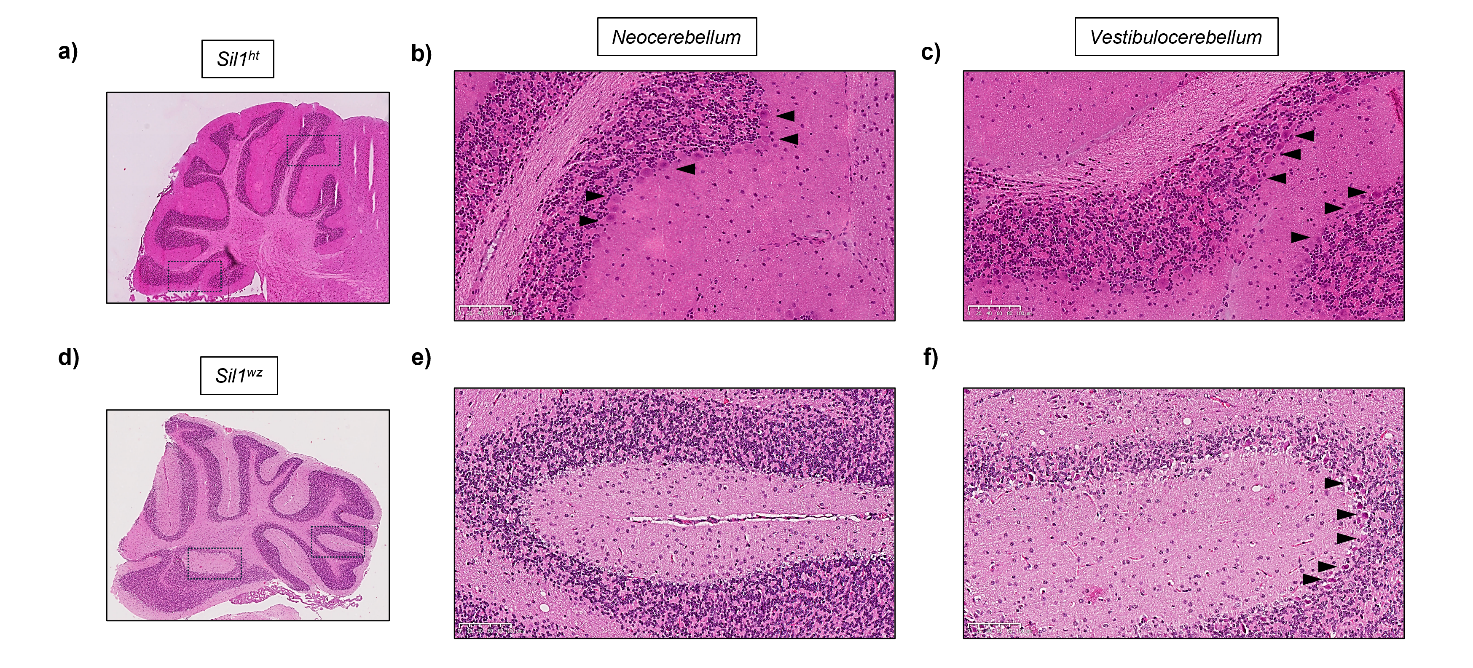


**Supplementary Table 1**:

| **Ab** | **Company** | **Identification code** | **Molecular weight (kDa)** | **Source** | **Diluition used** |
| --- | --- | --- | --- | --- | --- |
| BiP/GRP78 | BD Bioscience | 610979 | 78 | Mouse | 1:1,000 |
| P4HB/PDI | Abcam | Ab2792 | 57 | Mouse | 1:1,000 |
| eIF2α | SantaCruz | Sc-11386 | 38 | Rabbit | 1:800 |
| Phospho-eIF2α | Cell Signaling | 9721 | 38 | Rabbit | 1:500 |
| Chop/GADD153 | SantaCruz | Sc-7351 | 30 | Rabbit | 1:1,000 |
| Rab11 | BD Bioscience | 610657 | 24 | Mouse | 1:500 |
| LC3 | Novus Biologicals - Biotechne | NB100-2220 | 15 | Rabbit | 1:1,000 |
| CkB | Abcam | Ab92452 | 43 | Rabbit | 1:5,000 |
| Gapdh | SantaCruz | Sc-32233 | 38 | Mouse | 1:2,000 |

**Supplementary Table 2**:

|  | Mean (% ± SEM) | Mean (% ± SEM) | p value |  |
| --- | --- | --- | --- | --- |
| *Overall* |  |  |  |  |
|  | | | |  |
| Week | *Sil1^ht^* | *Sil1^wz^* |  |  |
| 5 | 100 ± 0.00 | 100 ± 0.00 | >0.9999 |  |
| 6 | 85.12 ± 4.45 | 87.49 ± 4.46 | 0.8742 |  |
| 7 | 87.22 ± 5.33 | 77.77 ± 4.40 | 0.1322 |  |
| 8 | 93.03 ± 2.75 | 74.95 ± 5.99 | 0.1322 |  |
| 9 | 88.09 ± 4.11 | 65.55 ± 4.54 | **0.0182** |  |
| 10 | 87.91 ± 3.80 | 55.59 ± 6.02 | **0.0024** |  |
| 11 | 89.91 ± 4.74 | 49.35 ± 5.86 | **0.0004** |  |
| 12 | 92.30 ± 3.46 | 42.73 ± 6.14 | **< 0.0001** |  |
| 13 | 93.85 ± 3.08 | 35.23 ± 5.41 | **< 0.0001** |  |
| 14 | 93.79 ± 3.07 | 32.37 ± 5.59 | **< 0.0001** |  |
| *Males* | | | |  |
|  |  | |  |  |
| Week | *Sil1^ht^* | *Sil1^wz^* |  |  |
| 5 | 100 ± 0.00 | 100 ± 0.00 | >0.9999 |  |
| 6 | 81.36 ± 5.90 | 86.24 ± 8.68 | 0.5469 |  |
| 7 | 89.42 ± 4.92 | 73.78 ± 7.37 | 0.3313 |  |
| 8 | 95.24 ± 3.73 | 62.74 ± 5.79 | **0.0426** |  |
| 9 | 89.73 ± 6.03 | 59.23 ± 3.61 | **0.0426** |  |
| 10 | 90.61 ± 4.16 | 47.53 ± 8.35 | **0.0426** |  |
| 11 | 90.87 ± 7.17 | 34.67 ± 5.46 | **0.0426** |  |
| 12 | 94.64 ± 4.75 | 30.50 ± 7.02 | **0.0426** |  |
| 13 | 91.15 ± 6.23 | 22.43 ± 2.98 | **0.0426** |  |
| 14 | 89.25 ± 6.14 | 20.72 ± 4.25 | **0.0426** |  |
| *Females* | | | |  |
|  |  | |  |  |
| Week | *Sil1^ht^* | *Sil1^wz^* |  |  |
| 5 | 100 ± 0.00 | 100 ± 0.00 | >0.9999 |  |
| 6 | 88.26 ± 6.7 | 88.75 ± 3.39 | 0.9745 |  |
| 7 | 85.39 ± 9.31 | 81.76 ± 4.96 | 0.6553 |  |
| 8 | 91.19 ± 4.32 | 87.16 ± 8.04 | 0.9958 |  |
| 9 | 86.72 ± 6.07 | 71.87 ± 7.85 | 0.6524 |  |
| 10 | 85.66 ± 6.26 | 63.66 ± 7.99 | 0.3316 |  |
| 11 | 89.10 ± 6.92 | 64.03 ± 5.93 | 0.1552 |  |
| 12 | 90.34 ± 5.20 | 54.96 ± 7.55 | **0.0341** |  |
| 13 | 96.10 ± 5.60 | 48.04 ± 7.39 | **0.0214** |  |
| 14 | 97.57 ± 1.76 | 44.02 ± 8.08 | **0.0214** |  |
| *Sil1^ht^* | | | | |
|  | |  | |  |
| Week | | *Males* | *Females* |  |
| 5 | | 100 ± 0.00 | 100 ± 0.00 | >0.9999 |
| 6 | | 81.36 ± 5.90 | 88.26 ± 6.7 | 0.9809 |
| 7 | | 89.42 ± 4.92 | 85.39 ± 9.31 | 0.9965 |
| 8 | | 95.24 ± 3.37 | 91.19 ± 4.32 | 0.9965 |
| 9 | | 89.73 ± 6.03 | 86.72 ± 6.07 | 0.9965 |
| 10 | | 90.61 ± 4.16 | 85.66 ± 6.26 | 0.9960 |
| 11 | | 90.87 ± 7.17 | 89.10 ± 6.92 | 0.9951 |
| 12 | | 94.64 ± 4.75 | 90.34 ± 5.20 | 0.9965 |
| 13 | | 91.15 ± 6.23 | 96.10 ± 5.60 | 0.9965 |
| 14 | | 89.25 ± 6.14 | 97.57 ± 1.76 | 0.9960 |
| *Sil1^wz^* | | | | |
|  | |  | |  |
| Week | | *Males* | *Females* |  |
| 5 | | 100 ± 0.00 | 100 ± 0.00 | >0.9999 |
| 6 | | 86.24 ± 8.68 | 88.75 ± 3.39 | 0.8448 |
| 7 | | 73.78 ± 7.37 | 81.76 ± 4.96 | 0.8431 |
| 8 | | 62.74 ± 5.79 | 87.16 ± 8.04 | 0.3007 |
| 9 | | 59.23 ± 3.61 | 71.87 ± 7.85 | 0.8431 |
| 10 | | 47.53 ± 8.35 | 63.66 ± 7.99 | 0.8431 |
| 11 | | 34.67 ± 5.46 | 64.03 ± 5.93 | **0.0425** |
| 12 | | 30.50 ± 7.02 | 54.96 ± 7.55 | 0.3007 |
| 13 | | 22.43 ± 2.98 | 48.04 ± 7.39 | 0.3007 |
| 14 | | 20.72 ± 4.25 | 44.02 ± 8.08 | 0.3007 |

**Supplementary Table 3**:

|  | | Mean (s ± SEM) | | p value | | Mean (n ± SEM) | | | p value |  |
| --- | --- | --- | --- | --- | --- | --- | --- | --- | --- | --- |
| *Overall* | | | | |  | | | | |  |
|  | *Time* | | |  | *Misstep* | | | |  |  |
| Week | *Sil1^ht^* | | *Sil1^wz^* |  | *Sil1^ht^* | | *Sil1^wz^* | |  |  |
| 5 | 4.72 ± 0.75 | | 6.74 ± 0.93 | 0.1329 | 0.65 ± 0.17 | | 1.18 ± 0.15 | | **0.0150** |  |
| 6 | 4.44 ± 0.83 | | 6.90 ± 1.13 | 0.1329 | 0.48 ± 0.12 | | 2.45 ± 0.51 | | **0.0002** |  |
| 7 | 4.00 ± 0.62 | | 8.03 ± 1.11 | **0.0208** | 0.70 ± 0.21 | | 4.58 ± 0.62 | | **< 0.0001** |  |
| 8 | 3.81 ± 0.55 | | 8.33 ± 0.73 | **0.0007** | 0.82 ± 0.23 | | 8.06 ± 0.77 | | **< 0.0001** |  |
| 9 | 3.76 ± 0.71 | | 13.18 ± 2.42 | **0.0007** | 0.70 ± 0.14 | | 9.64 ± 0.82 | | **< 0.0001** |  |
| 10 | 3.78 ± 0.61 | | 15.36 ± 2.53 | **< 0.0001** | 0.82 ± 0.14 | | 11.58 ± 0.67 | | **< 0.0001** |  |
| 11 | 3.96 ± 0.58 | | 16.75 ± 1.75 | **< 0.0001** | 0.79 ± 0.10 | | 15.31 ± 0.97 | | **< 0.0001** |  |
| 12 | 4.04 ± 0.54 | | 18.08 ± 1.45 | **< 0.0001** | 0.76 ± 0.20 | | 19.81 ± 1.30 | | **< 0.0001** |  |
| *Males* | | | | | | | | | |  |
|  | *Time* | | |  | *Misstep* | | | |  |  |
| Week | *Sil1^ht^* | | *Sil1^wz^* |  | *Sil1^ht^* | | | *Sil1^wz^* |  |  |
| 5 | 3.33 ± 0.22 | | 6.78 ± 0.75 | **0.0341** | 0.90 ± 0.28 | | | 1.36 ± 0.17 | 0.1710 |  |
| 6 | 2.92 ± 0.47 | | 5.52 ± 0.63 | **0.0426** | 0.47 ± 0.17 | | | 1.89 ± 0.32 | **0.0341** |  |
| 7 | 2.79 ± 0.39 | | 9.40 ± 1.87 | **0.0511** | 0.40 ± 0.20 | | | 4.72 ± 1.00 | **0.0341** |  |
| 8 | 3.39 ± 0.71 | | 8.83 ± 1.41 | **0.0511** | 0.80 ± 0.37 | | | 7.39 ± 1.18 | **0.0341** |  |
| 9 | 3.68 ± 1.42 | | 11.14 ± 1.89 | **0.0511** | 0.87 ± 0.25 | | | 8.44 ± 0.89 | **0.0341** |  |
| 10 | 3.76 ± 1.06 | | 14.93 ± 3.51 | **0.0426** | 0.67 ± 0.24 | | | 10.44 ± 0.74 | **0.0341** |  |
| 11 | 3.69 ± 0.56 | | 15.67 ± 2.35 | **0.0341** | 0.73 ± 0.16 | | | 16.17 ± 1.58 | **0.0341** |  |
| 12 | 3.82 ± 0.59 | | 17.17 ± 1.42 | **0.0341** | 1.00 ± 0.33 | | | 19.33 ± 1.69 | **0.0341** |  |
| *Females* | | | | | | | | | |  |
|  | *Time* | | |  | *Misstep* | | | |  |  |
| Week | *Sil1^ht^* | | *Sil1^wz^* |  | *Sil1^ht^* | | | *Sil1^wz^* |  |  |
| 5 | 5.88 ± 1.21 | | 6.70 ± 1.80 | > 0.9999 | 0.43 ± 0.16 | | | 1.00 ± 0.23 | **0.0427** |  |
| 6 | 5.71 ± 1.29 | | 8.28 ± 2.10 | 0.7774 | 0.57 ± 0.17 | | | 3.00 ± 0.96 | **0.0427** |  |
| 7 | 5.00 ± 0.93 | | 6.67 ± 1.09 | 0.7774 | 0.85 ± 0.29 | | | 4.45 ± 0.83 | **0.0070** |  |
| 8 | 4.15 ± 0.86 | | 7.83 ± 0.50 | 0.0999 | 0.81 ± 0.27 | | | 8.72 ± 1.01 | **0.0070** |  |
| 9 | 3.83 ± 0.69 | | 15.22 ± 4.53 | **0.0426** | 0.66 ± 0.16 | | | 10.83 ± 1.26 | **0.0070** |  |
| 10 | 3.79 ± 0.77 | | 15.79 ± 3.97 | **0.0257** | 0.86 ± 0.16 | | | 12.72 ± 0.93 | **0.0047** |  |
| 11 | 4.18 ± 1.00 | | 17.83 ± 2.73 | **0.0172** | 1.00 ± 0.21 | | | 14.44 ± 1.16 | **0.0070** |  |
| 12 | 4.23 ± 0.91 | | 18.99 ± 2.62 | **0.0172** | 0.67 ± 0.22 | | | 20.28 ± 2.12 | **0.0047** |  |
| *Sil1^ht^* | | | | | | | | | | |
|  | | *Time* | | |  | *Misstep* | | | |  |
| Week | | *Males* | | *Females* |  | *Males* | | | *Females* |  |
| 5 | | 3.33 ± 0.22 | | 5.88 ± 1.21 | 0.6581 | 0.90 ± 0.28 | | | 0.44 ± 0.19 | 0.1606 |
| 6 | | 2.92 ± 0.47 | | 5.71 ± 1.29 | 0.8174 | 0.47 ± 0.17 | | | 0.50 ± 0.19 | >0.9999 |
| 7 | | 2.79 ± 0.39 | | 5.00 ± 0.93 | 0.6581 | 0.40 ± 0.20 | | | 0.94 ± 0.33 | 0.8289 |
| 8 | | 3.39 ± 0.71 | | 4.15 ± 0.86 | 0.9870 | 0.80 ± 0.37 | | | 0.83 ± 0.32 | >0.999 |
| 9 | | 3.68 ± 1.42 | | 3.83 ± 0.69 | 0.9787 | 0.87 ± 0.25 | | | 0.55 ± 0.14 | 0.9070 |
| 10 | | 3.76 ± 1.06 | | 3.79 ± 0.77 | 0.9952 | 0.67 ± 0.24 | | | 0.94 ± 0.16 | 0.9107 |
| 11 | | 3.69 ± 0.56 | | 4.18 ± 1.00 | 0.9952 | 0.73 ± 0.16 | | | 0.83 ± 0.14b | 0.9883 |
| 12 | | 3.82 ± 0.59 | | 4.23 ± 0.91 | 0.9910 | 1.00 ± 0.33 | | | 0.55 ± 0.22 | 0.9107 |
| *Sil1^wz^* | | | | | | | | | | |
|  | | *Time* | | |  | *Misstep* | | | |  |
| Week | | *Males* | | *Females* |  | *Males* | | | *Females* |  |
| 5 | | 6.78 ± 0.75 | | 6.70 ± 1.80 | 0.9918 | 1.36 ± 0.17 | | | 1.00 ± 0.23 | 0.7421 |
| 6 | | 5.52 ± 0.63 | | 8.28 ± 2.10 | 0.9818 | 1.89 ± 0.32 | | | 3.00 ± 0.96 | 0.9296 |
| 7 | | 9.40 ± 1.87 | | 6.67 ± 1.09 | 0.9818 | 4.72 ± 1.00 | | | 4.45 ± 0.83 | 0.9296 |
| 8 | | 8.83 ± 1.41 | | 7.83 ± 0.50 | 0.9818 | 7.39 ± 1.18 | | | 8.72 ± 1.01 | 0.8224 |
| 9 | | 11.14 ± 1.89 | | 15.22 ± 4.53 | 0.9940 | 8.44 ± 0.89 | | | 10.83 ± 1.26 | 0.7421 |
| 10 | | 14.93 ± 3.51 | | 15.79 ± 3.97 | 0.9940 | 10.44 ± 0.74 | | | 12.72 ± 0.93 | 0.7421 |
| 11 | | 15.67 ± 2.35 | | 17.83 ± 2.73 | 0.9818 | 16.17 ± 1.58 | | | 14.44 ± 1.16 | 0.9296 |
| 12 | | 17.17 ± 1.42 | | 18.99 ± 2.62 | 0.9940 | 19.33 ± 1.69 | | | 20.28 ± 2.12 | 0.9296 |

**Supplementary Table 4**:

|  | | Mean (s ± SEM) | | p value | | Mean (s ± SEM) | | | p value |  |
| --- | --- | --- | --- | --- | --- | --- | --- | --- | --- | --- |
| *Overall* | | | | |  | | | | |  |
|  | *T-turn* | | |  | *T-total* | | | |  |  |
| Week | *Sil1^ht^* | | *Sil1^wz^* |  | *Sil1^ht^* | | *Sil1^wz^* | |  |  |
| 14 | 3.42 ± 1.05 | | 7.55 ± 2.19 | **0.0090** | 9.87 ± 3.72 | | 14.02 ± 2.72 | | **0.0129** |  |
| 16 | 2.70 ± 0.82 | | 4.81 ± 0.55 | **0.0009** | 9.09 ± 1.96 | | 13.88 ±1.91 | | **0.0111** |  |
| *Males* | | | | | | | | | |  |
|  | *T-turn* | | |  | *T-total* | | | |  |  |
| Week | *Sil1^ht^* | | *Sil1^wz^* |  | *Sil1^ht^* | | | *Sil1^wz^* |  |  |
| 14 | 2.17 ± 0.31 | | 11.72 ± 3.76 | **0.0086** | 7.23 ± 1.05 | | | 20.04 ± 4.22 | **0.0086** |  |
| 16 | 1.89 ± 0.24 | | 5.95 ± 0.77 | **0.0086** | 6.35 ± 0.58 | | | 15.32 ± 1.76 | **0.0086** |  |
| *Females* | | | | | | | | | |  |
|  | *T-turn* | | |  | *T-total* | | | |  |  |
| Week | *Sil1^ht^* | | *Sil1^wz^* |  | *Sil1^ht^* | | | *Sil1^wz^* |  |  |
| 14 | 4.45 ± 1.86 | | 3.38 ± 0.28 | 0.0589 | 12.06 ± 6.92 | | | 8.00 ± 0.61 | 0.2466 |  |
| 16 | 3.37 ± 1.49 | | 3.67 ± 0.45 | 0.1378 | 11.36 ± 3.41 | | | 12.44 ± 3.48 | 0.6191 |  |
| *Sil1^ht^* | | | | | | | | | | |
|  | | *T-turn* | | |  | *T-total* | | | |  |
| Week | | *Males* | | *Females* |  | *Males* | | | *Females* |  |
| 14 | | 2.17 ± 0.31 | | 4.45 ± 1.86 | 0.4326 | 7.23 ± 1.05 | | | 12.06 ± 6.92 | 0.5368 |
| 16 | | 1.89 ± 0.24 | | 3.37 ± 1.49 | 0.6623 | 6.35 ± 0.58 | | | 11.36 ± 3.41 | 0.1577 |
| *Sil1^wz^* | | | | | | | | | | |
|  | | *T-turn* | | |  | *T-total* | | | |  |
| Week | | *Males* | | *Females* |  | *Males* | | | *Females* |  |
| 14 | | 11.72 ± 3.76 | | 3.38 ± 0.28 | **0.0043** | 20.04 ± 4.22 | | | 8.00 ± 0.61 | **0.0043** |
| 16 | | 5.95 ± 0.77 | | 3.67 ± 0.45 | **0.0455** | 15.32 ± 1.76 | | | 12.44 ± 3.48 | 0.2403 |

**Supplementary Table 5**:

| Time (s) | | | | | | | | | | | | | | | |
| --- | --- | --- | --- | --- | --- | --- | --- | --- | --- | --- | --- | --- | --- | --- | --- |
|  | | Mean (s ± SEM) | | | | | p value | | Mean (s ± SEM) | | | | | p value | |
|  | | ***Latency to fall (s)*** | | | | |  | | ***Immobility (s)*** | | | | |  | |
| *Overall* | |  | | | | |  | |  | | | | |  | |
|  | | *Sil1^ht^* | | *Sil1^wz^* | | |  | | *Sil1^ht^* | | | *Sil1^wz^* | |  | |
|  | | 106.70 ± 9.70 | | 102.50 ± 9.80 | | | 0.06476 | | 9.87 ± 3.72 | | | 14.02 ± 2.72 | | **0.0129** | |
| *Males* | | | | | | | | | | | | | | | |
|  | | *Sil1^ht^* | | *Sil1^wz^* | | |  | | *Sil1^ht^* | | | *Sil1^wz^* | |  | |
|  | | 88.11 ± 21.74 | | 104.90 ± 15.09 | | | 0.4242 | | 0.00 ± 0.00 | | | 7.59 ± 3.12 | | **0.0152** | |
| *Females* | | | | | | | | | | | | | | | |
|  | | *Sil1^ht^* | | *Sil1^wz^* | | |  | | *Sil1^ht^* | | | *Sil1^wz^* | |  | |
|  | | 103.00 ± 11.54 | | 120.00 ± 0.00 | | | 0.4545 | | 0.95 ± 0.64 | | | 8.26 ± 2.38 | | **0.0216** | |
| *Sil1^ht^* | |  | |  | | |  | |  | | |  | |  | |
|  | | *Males* | | *Females* | | |  | | *Males* | | | *Females* | |  | |
|  | | 88.11 ± 21.74 | | 103.00 ± 11.54 | | | 0.1818 | | 0.00 ± 0.00 | | | 0.95 ± 0.64 | | 0.4545 | |
| *Sil1^wz^* | |  | |  | | |  | |  | | |  | |  | |
|  | | *Males* | | *Females* | | |  | | *Males* | | | *Females* | |  | |
|  | | 104.90 ± 15.09 | | 120.00 ± 0.00 | | | >0.9999 | | 7.59 ± 3.12 | | | 8.26 ± 2.38 | | 0.6234 | |
| Time (n) | | | | | | | | | | | | | | | |
|  | Mean (n ± SEM) | | | | p value | Mean (n ± SEM) | | | | p value | Mean (n ± SEM) | | | | p value |
|  | ***Immobility (n)*** | | | |  | ***Forelimbs (n)*** | | | |  | ***Hindlimbs (n)*** | | | |  |
| *Overall* |  | | | |  |  | | | |  |  | | | |  |
|  | *Sil1^ht^* | | *Sil1^wz^* | |  | *Sil1^ht^* | | *Sil1^wz^* | |  | *Sil1^ht^* | | *Sil1^wz^* | |  |
|  | 0.18 ± 0.12 | | 3.08 ± 0.60 | | **0.0007** | 2.64 ± 0.81 | | 1.17 ± 0.56 | | 0.1221 | 3.46 ± 0.84 | | 0.17 ± 0.11 | | **0.0009** |
| *Males* | | | | | | | | | | | | | | | |
|  | *Sil1^ht^* | | *Sil1^wz^* | |  | *Sil1^ht^* | | *Sil1^wz^* | |  | *Sil1^ht^* | | *Sil1^wz^* | |  |
|  | 0.00 ± 0.00 | | 3.17 ± 0.95 | | **0.0448** | 2.00 ± 0.84 | | 1.00 ± 0.68 | | 0.4048 | 4.20 ± 1.56 | | 1.17 ± 0.17 | | 0.0555 |
| *Females* | | | | | | | | | | | | | | | |
|  | *Sil1^ht^* | | *Sil1^wz^* | |  | *Sil1^ht^* | | *Sil1^wz^* | |  | *Sil1^ht^* | | *Sil1^wz^* | |  |
|  | 3.00 ± 0.82 | | 0.33 ± 0.21 | | 0.0635 | 3.17 ± 1.35 | | 1.33 ± 0.96 | | 0.2619 | 2.83 ± 0.87 | | 0.17 ± 0.17 | | 0.0635 |
| *Sil1^ht^* |  | |  | |  |  | |  | |  |  | |  | |  |
|  | *Males* | | *Females* | |  | *Males* | | *Females* | |  | *Males* | | *Females* | |  |
|  | 0.00 ± 0.00 | | 3.00 ± 0.82 | | 0.8377 | 2.00 ± 0.84 | | 3.17 ± 1.35 | | 0.8377 | 4.20 ± 1.56 | | 2.83 ± 0.87 | | 0.8377 |
| *Sil1^wz^* |  | |  | |  |  | |  | |  |  | |  | |  |
|  | *Males* | | *Females* | |  | *Males* | | *Females* | |  | *Males* | | *Females* | |  |
|  | 3.17 ± 0.95 | | 0.33 ± 0.21 | | 0.9995 | 1.00 ± 0.68 | | 1.33 ± 0.96 | | 0.9965 | 1.17 ± 0.17 | | 0.17 ± 0.17 | | >0.9999 |

**Supplementary Table 6**:

|  | Mean (s ± SEM) | | p value | Mean (s ± SEM) | | p value | Mean (s ± SEM) | | p value | Mean (s ± SEM) | | p value |
| --- | --- | --- | --- | --- | --- | --- | --- | --- | --- | --- | --- | --- |
|  | ***Movement*** | | | ***Opening*** | | | ***Gnawing*** | | | ***Total*** | | |
| *Overall* |  | |  |  | |  |  | |  |  |  | |
| Week | *Sil1^ht^* | *Sil1^wz^* |  | *Sil1^ht^* | *Sil1^wz^* |  | *Sil1^ht^* | *Sil1^wz^* |  | *Sil1^ht^* | *Sil1^wz^* |  |
| 10 | 2.64 ± 0.38 | 3.41 ± 0.38 | 0.3876 | 1.71 ± 0.15 | 1.37 ± 0.19 | 0.3404 | 2.70 ± 0.36 | 3.03 ± 0.41 | 0.5413 | 2.35 ± 0.18 | 2.60 ± 0.20 | 0.3084 |
| 14 | 2.92 ± 0.39 | 2.43 ± 0.33 | 0.5413 | 2.06 ± 0.14 | 1.58 ± 0.16 | 0.3027 | 3.08 ± 0.25 | 1.79 ± 0.25 | **0.0120** | 2.69 ± 0.15 | 1.94 ± 0.19 | **0.0199** |
|  | | | | | | | | | | | | |
| *Males* |  | |  |  | |  |  |  |  |  |  |  |
| Week | *Sil1^ht^* | *Sil1^wz^* |  | *Sil1^ht^* | *Sil1^wz^* |  | *Sil1^ht^* | *Sil1^wz^* |  | *Sil1^ht^* | *Sil1^wz^* |  |
| 10 | 3.10 ± 0.66 | 3.92 ± 0.35 | 0.7821 | 1.60 ± 0.27 | 1.21 ± 0.14 | 0.7236 | 1.73 ± 0.32 | 2.44 ± 0.66 | 0.7822 | 2.14 ± 0.19 | 2.52 ± 0.30 | 0.3528 |
| 14 | 3.23 ± 0.62 | 2.46 ± 0.55 | 0.7823 | 2.00 ± 0.21 | 1.33 ± 0.21 | 0.2639 | 2.67 ± 0.38 | 1.47 ± 0.33 | 0.2685 | 2.63 ± 0.26 | 1.76 ± 0.32 | 0.1814 |
|  | | | | | | | | | | | | |
| *Females* |  | |  |  | |  |  |  |  |  |  |  |
| Week | *Sil1^ht^* | *Sil1^wz^* |  | *Sil1^ht^* | *Sil1^wz^* |  | *Sil1^ht^* | *Sil1^wz^* |  | *Sil1^ht^* | *Sil1^wz^* |  |
| 10 | 2.25 ± 0.40 | 2.90 ± 0.63 | 0.9488 | 1.81 ± 0.19 | 1.53 ± 0.35 | 0.9488 | 3.50 ± 0.37 | 3.61 ± 0.40 | 0.9488 | 2.52 ± 0.28 | 2.68 ± 0.30 | 0.6147 |
| 14 | 2.67 ± 0.52 | 2.40 ± 0.41 | 0.9488 | 2.11 ± 0.20 | 1.83 ± 0.21 | 0.9488 | 3.42 ± 0.28 | 2.11 ± 0.34 | 0.0875 | 2.73 ± 0.20 | 2.12 ± 0.22 | 0.1257 |
|  | | | | | | | | | | | | |
| *Sil1^ht^* |  | |  |  | |  |  |  |  |  |  |  |
| Week | *Males* | *Females* |  | *Males* | *Females* |  | *Males* | *Females* |  | *Males* | *Females* |  |
| 10 | 3.10 ± 0.66 | 2.25 ± 0.40 | 0.8885 | 1.60 ± 0.27 | 1.81 ± 0.19 | 0.9368 | 1.73 ± 0.32 | 3.50 ± 0.37 | 0.0508 | 2.14 ± 0.19 | 2.52 ± 0.28 | 0.5498 |
| 14 | 3.23 ± 0.62 | 2.67 ± 0.52 | 0.9368 | 2.00 ± 0.21 | 2.11 ± 0.20 | 0.9368 | 2.67 ± 0.38 | 3.42 ± 0.28 | 0.5822 | 2.63 ± 0.26 | 2.73 ± 0.20 | 0.7922 |
|  | | | | | | | | | | | | |
| *Sil1^wz^* |  | |  |  | |  |  |  |  |  |  |  |
| Week | *Males* | *Females* |  | *Males* | *Females* |  | *Males* | *Females* |  | *Males* | *Females* |  |
| 10 | 3.92 ± 0.35 | 2.90 ± 0.63 | 0.7527 | 1.21 ± 0.14 | 1.53 ± 0.35 | 0.9081 | 2.44 ± 0.66 | 3.61 ± 0.40 | 0.6477 | 2.52 ± 0.30 | 2.68 ± 0.30 | 0.6688 |
| 14 | 2.46 ± 0.55 | 2.40 ± 0.41 | 0.9935 | 1.33 ± 0.21 | 1.83 ± 0.21 | 0.6477 | 1.47 ± 0.33 | 2.11 ± 0.34 | 0.4109 | 1.76 ± 0.32 | 2.12 ± 0.22 | 0.4960 |

**Supplementary Table 7**:

| *Discrimination index (%)* | | | |  |
| --- | --- | --- | --- | --- |
|  | Mean (s ± SEM) | | p value |  |
| *Overall* |  | |  |  |
|  |  | |  |  |
| Week | *Sil1^ht^* | *Sil1^wz^* |  |  |
| 5 | 66.03 ± 5.02 | 59.08 ± 7.06 | 0.7780 |  |
| 14 | 66.48 ± 7.67 | 62.53 ± 8.07 | 0.7780 |  |
| *Males* | | | |  |
|  |  | |  |  |
| Week | *Sil1^ht^* | *Sil1^wz^* |  |  |
| 5 | 64.50 ± 8.51 | 53.78 ± 6.90 | 0.5682 |  |
| 14 | 81.63 ± 5.29 | 73.43 ± 7.72 | 0.5682 |  |
| *Females* | | | |  |
|  |  | |  |  |
| Week | *Sil1^ht^* | *Sil1^wz^* |  |  |
| 5 | 68.34 ± 2.29 | 67.01 ± 14.81 | 0.5682 |  |
| 14 | 56.38 ± 10.68 | 55.27 ± 12.06 | 0.9372 |  |
| *Sil1^ht^* | | | | |
|  | |  | |  |
| Week | | *Males* | *Females* |  |
| 5 | | 64.50 ± 8.51 | 68.34 ± 2.29 | >0.9999 |
| 14 | | 81.63 ± 5.29 | 56.38 ± 10.68 | 0.4482 |
| *Sil1^wz^* | | | | |
|  | |  | |  |
| Week | | *Males* | *Females* |  |
| 5 | | 53.78 ± 6.90 | 67.01 ± 14.81 | 0.4482 |
| 14 | | 73.43 ± 7.72 | 55.27 ± 12.06 | 0.4482 |

**Supplementary Table 8**:

| *Arms alternation (%)* | | | | | |  |
| --- | --- | --- | --- | --- | --- | --- |
|  | | Mean (s ± SEM) | | | p value |  |
| *Overall* | | | |  | |  |
|  |  | | | |  |  |
| Week | *Sil1^ht^* | | *Sil1^wz^* | |  |  |
| 5 | 68.00 ± 2.62 | | 69.60 ± 4.20 | | 0.9639 |  |
| 14 | 66.00 ± 3.06 | | 67.30 ± 2.93 | | 0.9639 |  |
| *Males* | | | | | |  |
|  | *T-turn* | | | |  |  |
| Week | *Sil1^ht^* | | *Sil1^wz^* | |  |  |
| 5 | 63.67 ± 3.03 | | 66.17 ± 5.14 | | 0.6753 |  |
| 14 | 62.25 ± 5.54 | | 70.25 ± 1.84 | | 0.5298 |  |
| *Females* | | | | | |  |
|  | *T-turn* | | | |  |  |
| Week | *Sil1^ht^* | | *Sil1^wz^* | |  |  |
| 5 | 74.50 ± 2.18 | | 74.75 ± 7.18 | | >0.9999 |  |
| 14 | 68.50 ± 3.56 | | 65.33 ± 4.73 | | 0.9095 |  |
| *Sil1^ht^* | | | | | | |
|  | | *T-turn* | | | |  |
| Week | | *Males* | | *Females* | |  |
| 5 | | 63.67 ± 3.03 | | 74.50 ± 2.18 | | 0.1289 |
| 14 | | 62.25 ± 5.54 | | 68.50 ± 3.56 | | 0.6857 |
| *Sil1^wz^* | | | | | | |
|  | | *T-turn* | | | |  |
| Week | | *Males* | | *Females* | |  |
| 5 | | 66.17 ± 5.14 | | 74.75 ± 7.18 | | 0.6109 |
| 14 | | 70.25 ± 1.84 | | 65.33 ± 4.73 | | 0.6109 |

**Supplementary Table 9**:

| Overall | | | | | | | | | | |  |
| --- | --- | --- | --- | --- | --- | --- | --- | --- | --- | --- | --- |
|  | **Rotarod (%)** | **BW Time** | **BW steps** | **IS Immobility (s)** | **IS Immobility (n)** | **IS Forelimbs** | **IS Hindlimbs** | **PT Total** | **PT Turn** | **NB Ave** | |
| Rotarod (%) |  | -0.706 | -0.694 | -0.536 | -0.629 | 0.305 | 0.633 | -0.358 | -0.544 | 0.538 | |
| BW Time | **0.0002** |  | 0.772 | 0.775 | 0.723 | -0.234 | -0.630 | 0.490 | 0.565 | -0.547 | |
| BW steps | **0.0002** | **<0.0001** |  | 0.780 | 0.815 | -0.262 | -0.726 | 0.621 | 0.637 | -0.416 | |
| IS Immobility (s) | **0.008** | **<0.0001** | **<0.0001** |  | 0.948 | -0.292 | -0.587 | 0.555 | 0.491 | -0.332 | |
| IS Immobility (n) | **0.001** | **<0.0001** | **<0.0001** | **<0.0001** |  | -0.245 | -0.614 | 0.611 | 0.594 | -0.351 | |
| IS Forelimbs | 0.157 | 0.283 | 0.228 | 0.176 | 0.261 |  | 0.350 | 0.013 | -0.135 | 0.077 | |
| IS Hindlimbs | **0.001** | **0.001** | **<0.0001** | **0.003** | **0.002** | 0.102 |  | -0.446 | -0.523 | 0.370 | |
| PT Total | **0.093** | **0.018** | **0.002** | **0.006** | **0.002** | 0.955 | 0.033 |  | 0.848 | -0.420 | |
| PT Turn | **0.007** | **0.005** | **0.001** | **0.017** | **0.003** | 0.538 | **0.010** | **<0.0001** |  | -0.554 | |
| NB Ave | **0.008** | **0.007** | **0.049** | 0.122 | 0.101 | 0.726 | 0.082 | **0.046** | **0.006** |  | |

| *Sil1^ht^* | | | | | | | | | | |
| --- | --- | --- | --- | --- | --- | --- | --- | --- | --- | --- |
|  | **Rotarod (%)** | **BW Time** | **BW steps** | **IS Immobility (s)** | **IS Immobility (n)** | **IS Forelimbs** | **IS Hindlimbs** | **PT Total** | **PT Turn** | **NB Ave** |
| Rotarod (%) |  | 0.130 | 0.107 | 0.069 | 0.000 | 0.217 | -0.279 | 0.647 | 0.447 | 0.042 |
| BW Time | 0.701 |  | 0.214 | 0.351 | 0.100 | 0.279 | -0.138 | 0.482 | 0.118 | -0.209 |
| BW steps | 0.749 | 0.524 |  | 0.518 | 0.256 | -0.174 | -0.704 | 0.200 | -0.112 | 0.019 |
| IS Immobility (s) | 0.864 | 0.309 | 0.145 |  | 0.742 | -0.435 | -0.572 | 0.526 | 0.094 | -0.229 |
| IS Immobility (n) | 1.000 | 0.909 | 0.636 | 0.091 |  | -0.409 | -0.454 | 0.400 | 0.200 | -0.200 |
| IS Forelimbs | 0.515 | 0.402 | 0.610 | 0.236 | 0.364 |  | 0.014 | 0.288 | 0.023 | 0.042 |
| IS Hindlimbs | 0.399 | 0.686 | **0.019** | 0.073 | 0.273 | 0.968 |  | -0.486 | -0.170 | -0.156 |
| PT Total | **0.036** | 0.138 | 0.552 | 0.109 | 0.364 | 0.386 | 0.132 |  | 0.655 | -0.273 |
| PT Turn | 0.169 | 0.735 | 0.743 | 0.800 | 0.727 | 0.950 | 0.616 | **0.034** |  | -0.309 |
| NB Ave | 0.906 | 0.539 | 0.961 | 0.509 | 0.727 | 0.906 | 0.646 | 0.418 | 0.356 |  |

| *Sil1^wz^* | | | | | | | | | | |
| --- | --- | --- | --- | --- | --- | --- | --- | --- | --- | --- |
|  | **Rotarod (%)** | **BW Time** | **BW steps** | **IS Immobility (s)** | **IS Immobility (n)** | **IS Forelimbs** | **IS Hindlimbs** | **PT Total** | **PT Turn** | **NB Ave** |
| Rotarod (%) |  | 0.224 | 0.345 | 0.417 | 0.247 | -0.066 | 0.065 | -0.112 | -0.161 | 0.399 |
| BW Time | 0.485 |  | -0.172 | 0.634 | 0.165 | -0.102 | 0.194 | 0 | -0.228 | -0.172 |
| BW steps | 0.271 | 0.590 |  | 0.454 | 0.675 | 0.248 | 0.326 | 0.496 | 0.271 | 0.278 |
| IS Immobility (s) | 0.178 | **0.030** | 0.138 |  | 0.828 | 0.004 | 0.195 | 0.210 | -0.046 | 0.112 |
| IS Immobility (n) | 0.437 | 0.607 | **0.019** | **0.001** |  | 0.102 | 0 | 0.401 | 0.233 | 0.113 |
| IS Forelimbs | 0.840 | 0.752 | 0.433 | 0.994 | 0.754 |  | 0.217 | 0.070 | 0.033 | -0.235 |
| IS Hindlimbs | 0.909 | 0.606 | 0.318 | 0.591 | 1 | 0.576 |  | 0.194 | -0.195 | 0.195 |
| PT Total | 0.733 | 1 | 0.104 | 0.509 | 0.196 | 0.828 | 0.606 |  | 0.764 | -0.126 |
| PT Turn | 0.615 | 0.474 | 0.389 | 0.889 | 0.463 | 0.919 | 0.576 | **0.0057** |  | -0.351 |
| NB Ave | 0.198 | 0.592 | 0.376 | 0.726 | 0.723 | 0.457 | 0.591 | 0.695 | 0.261 |  |

**Supplementary Table 10**:

| **Protein** | **Quadriceps**  **Mean ± SEM** | |  | **Soleus**  **Mean ± SEM** | |  |
| --- | --- | --- | --- | --- | --- | --- |
|  | ***Sil1^ht^*** | ***Sil1^wz^*** | **P value** | ***Sil1^ht^*** | ***Sil1^wz^*** | **P value** |
| **BiP** | 0.99 ± 0.05 | 1.37 ± 0.06 | **0.0093** | 1.01 ± 0.03 | 0.97 ± 0.05 | 0.4558 |
| **EIF2α** | 0.51 ± 0.06 | 0.78 ± 0.13 | 0.1605 | 0.69 ± 0.04 | 0.75 ± 0.06 | 0.4545 |
| **pEIF2α** | 0.65 ± 0.21 | 1.51 ± 0.14 | **0.0279** | 0.71 ± 0.23 | 0.65 ± 0.10 | 0.8204 |
| **Rab11** | 0.16 ± 0.08 | 1.55 ± 0.17 | **0.0019** | 0.55 ± 0.27 | 1.09 ± 0.12 | 0.1384 |
| **Pdi** | 0.84 ± 0.08 | 1.14 ± 0.12 | 0.1090 | 0.93 ± 0.13 | 0.68 ± 0.14 | 0.2598 |
| **LC3-I** | 0.37 ± 0.08 | 0.65 ± 0.14 | 0.1601 | 0.36 ± 0.12 | 0.16 ± 0.05 | 0.2060 |
| **LC3-II** | 0.17 ± 0.01 | 0.67 ± 0.07 | **0.0022** | 0.34 ± 0.11 | 0.21 ± 0.04 | 0.3545 |
| **Chop** | 1.11 ± 0.14 | 1.80 ± 0.28 | 0.0939 | 0.57 ± 0.09 | 0.47 ± 0.22 | 0.7072 |
| **Ckb** | 0.94 ± 0.14 | 1.47 ± 0.25 | 0.1581 | 1.02 ± 0.04 | 0.95 ± 0.06 | 0.3270 |

**Supplementary Table 11**:

|  | **Mean ± SEM** | | **P value** |
| --- | --- | --- | --- |
|  | **Gastrocnemius** | |  |
|  | ***Sil1^ht^*** | ***Sil1^wz^*** |  |
| Males | 2188 ± 36.72 | 974.9 ± 14.91 | **< 0.0001** |
| Females | 1894 ± 28.94 | 973.7 ± 13.26 | **< 0.0001** |
|  | **Soleus** | |  |
|  | ***Sil1^ht^*** | ***Sil1^wz^*** |  |
| Males | 1468 ± 18.51 | 1471 ± 21.52 | 0.0993 |
| Females | 1479 ± 15.66 | 1499 ± 19.10 | 0.6558 |
